# Cost-Effective Voltammetric Determination of Salicylic Acid in Milk Using Copper Wire Electrodes

**DOI:** 10.1021/acsomega.5c06973

**Published:** 2025-10-10

**Authors:** Giulia C. P. Freitas, Vitoria B. Messias, Regina M. Takeuchi, André L. Santos

**Affiliations:** † 28119 Universidade Federal de Uberlândia, Instituto de Ciências Exatas e Naturais do Pontal, 38304-402 Ituiutaba, Brazil; ‡ Universidade Federal de Uberlândia, Instituto de Química, 38400-902 Uberlândia, Brazil

## Abstract

The illegal use of salicylic acid (SA) as a milk preservative raises significant health concerns, highlighting the need for accurate analytical methods capable of detecting this adulteration. This study presents an innovative, ecofriendly voltammetric method for SA determination in milk. The proposed method employs a working electrode fabricated from inexpensive, commercially available Cu wires, which were chemically modified with a CuO-rich layer grown electrochemically via cyclic voltammetry in alkaline medium (0.1 mol L^–1^ NaOH). This modifying layer not only enhanced the voltammetric response of SA but also conferred antifouling properties to the working electrode. Differential pulse voltammetry, under optimized conditions, yielded a linear range of 10–500 μmol L^–1^ with a limit of detection of 3.0 μmol L^–1^. A key innovation is a simple and rapid sample pretreatment using aqueous solutions of ZnSO_4_ and NaOH. The ZnSO_4_ precipitates proteins, while NaOH ionizes SA, facilitating its extraction into the aqueous phase. This procedure minimizes sample and reagent consumption, eliminates organic solvents, and allows the processing of up to eight samples in 15 min. This environmentally friendly pretreatment, combined with the Cu/CuO electrode, enabled reliable SA quantification in spiked milk samples, with recovery percentages ranging from 91% to 107%. This approach offers a cost-effective, sensitive, selective, and more sustainable method for SA quantification in milk.

## Graphical abstract

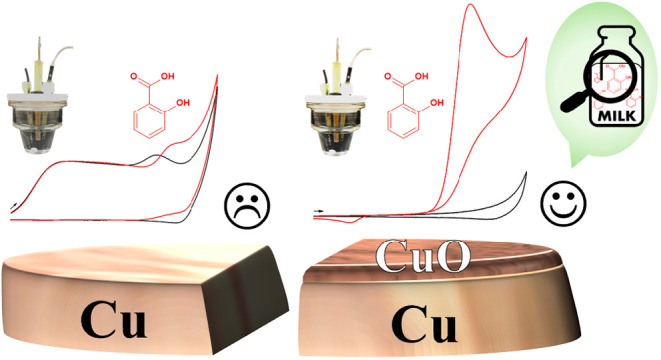

## 1 Introduction

Milk has long been recognized as a nutrient-rich food, providing significant amounts of calcium, potassium, a variety of vitamins, essential proteins, and beneficial fatty acids.^1,2^ Its high nutritional value has driven substantial growth in global production and consumption worldwide, particularly in developing countries such as Brazil. However, this rising demand has made milk a common target for adulteration,^2–4^ often with the intent to artificially enhance protein content or prolong shelf life. Chemicals frequently used for extending shelf life include formaldehyde, hydrogen peroxide, hypochlorite, and salicylic acid (SA).^2–6^ These substances pose serious risks to consumer health, making their deliberate addition to milk prohibited. As a primary ingredient in numerous food products, maintaining milk authenticity is critical to ensuring both consumer safety and the integrity of dairy-based products.

SA, 2-hydroxybenzoic acid according to IUPAC nomenclature, is a naturally occurring phenolic compound consisting of a benzene ring with ortho-positioned carboxyl (−COOH) and hydroxyl (−OH) groups. This structure confers unique properties, including moderate acidity (p*K*_a_ ≈ 3.0) and bioactive effects (antiseptic, anti-inflammatory, and exfoliating actions). SA serves as a precursor to acetylsalicylic acid and is widely used in pharmaceuticals, dermatological formulations, and cosmetics.^7^ Although prohibited for use in milk preservation, SA remains a frequently encountered adulterant, typically added at concentrations ranging from 0.04% to 0.05% (m/m).^8^ At these levels, consumption of adulterated milk can cause adverse health effects, including impaired digestibility, gastric irritation, bleeding, and diarrhea.^8,9^ Adulteration with SA is often performed with the assumption that detection will be unlikely, given the lack of standardized and accessible analytical methods. Therefore, the development of reliable and user-friendly procedures for quantifying SA in milk is essential to safeguard consumer health and ensure compliance with food safety regulations.

The A.O.A.C. Official Method 975.30 is widely used for detecting SA in food and beverages, including milk.^10^ It is a colorimetric method based on the formation of a purple complex between salicylate and Fe^3+^ ions. While effective, it provides only qualitative information and presents significant drawbacks when applied to milk analysis. It requires large sample volumes (∼25 mL) to ensure a detectable mass of SA and uses substantial amounts of diethyl ether (∼50 mL), a toxic organic solvent. Additionally, the procedure is labor-intensive and time-consuming, limiting its use in routine analysis.

Quantifying SA in milk is further complicated by strong noncovalent interactions between SA and proteins or fatty components in the sample matrix.^11,12^ To overcome these challenges, complex sample pretreatment procedures are often combined with more sophisticated instrumental techniques such as high-performance liquid chromatography (HPLC)^13^ and liquid chromatography coupled with ion trap mass spectrometry.^11,14,15^ While these methods provide high sensitivity and excellent detectability, they are costly and rely on complex instrumentation, restricting their accessibility.

Given the challenges associated with SA quantification in milk, the development of more practical, accessible, and environmentally friendly analytical methods for this purpose is of great relevance. Electroanalytical techniques have emerged as a promising alternative for food analysis^16,17^ particularly for milk samples.^18,19^ These techniques offer several advantages, including high sensitivity, instrumental simplicity, low cost, and the potential for real-time and on-site analysis. Furthermore, electroanalytical techniques often require minimal sample preparation and can be adapted for portable devices,^20–23^ making them suitable for routine analysis and field applications. While cyclic voltammetry (CV) and linear sweep voltammetry (LSV) are valuable for mechanistic studies and surface characterization, they are less suitable for quantitative analysis due to the influence of capacitive currents. In contrast, differential techniques such as differential pulse voltammetry (DPV) and square wave voltammetry (SWV) minimize capacitive contributions through pulsed potentials, providing higher sensitivity, better peak resolution, and lower detection limits.

A variety of electrodes has been developed for SA quantification, including bare glassy carbon electrode,^24^ aligned multiwalled carbon nanotube electrodes,^25^ carbon fiber electrodes,^26^ carbon paste electrodes chemically modified with zirconium oxide,^27^ screen-printed electrodes,^9^ graphite-epoxy composite electrodes,^28^ graphene-^29^ and reduced graphene-based^30^ electrodes, disposable stainless-steel wire electrodes,^31^ TiO_2_ nanorod arrays functionalized with molecularly imprinted polymers,^32^ and ZnO/CuO/Y_2_O_3_ nanocomposites embedded PEDOT-based electrodes.^33^ These electrodes have been successfully applied to quantify SA in diverse matrices, including pharmaceuticals, human serum, fruit juices, plant leaves, blood, and milk.

However, a significant challenge in electrochemical SA determination lies in electrode fouling, which occurs due to the formation of a polymeric film on the electrode surface during SA electrooxidation.^26,27^ This fouling significantly decreases the sensor’s sensitivity and reproducibility. To address this issue, various strategies have been proposed to remove the polymeric film from the electrode surface, such as anodic polarization and cleaning the electrode with strong bases.^26,27^

Copper electrodes are attractive for applications in electroanalysis due to their low cost, high electrical conductivity, and the ability to form an electrocatalytically active Cu­(OH)_2_/CuO surface layer in alkaline solutions.^34–37^ This layer plays a dual role: it acts as an electrocatalyst, enhancing the electrooxidation of various organic species, and provides a protective barrier against electrode fouling and corrosion.^37^ The electrocatalytic capabilities of copper electrodes have been explored for the electroanalytical determination of diverse analytes such as carbohydrates,^34,38^ alcohols,^39^ hydrazine,^40^ and sulfite.^41,42^ Therefore, the advantageous electrochemical properties of copper, combined with its affordability and widespread availability, highlight its potential as a reliable and efficient material for electrochemical sensing. Unlike other electrode materials that often require complex chemical modifications, Cu electrodes provide a straightforward, cost-effective alternative, with surface modification easily achieved through simple electrochemical protocols. Their ease of use, combined with electrocatalytic properties and antifouling properties, make Cu electrodes a superior choice for practical, large-scale electrochemical sensing, particularly in routine analysis where cost and simplicity are crucial.

To the best of our knowledge, no studies have reported the use of copper electrodes for the quantification of SA in milk samples. In this study, commercially available copper wires, typically used in domestic electrical installations, were employed as electrodes in alkaline media for the voltammetric determination of SA in bovine milk. After a simple electrochemical conditioning procedure, these electrodes provided a highly stable signal for SA, demonstrating notable antifouling properties. Additionally, a simple, rapid, and organic solvent-free sample pretreatment procedure was developed, requiring only 500 μL of milk sample and approximately 15 min to complete. This innovative approach enabled the development of a more sustainable, more efficient, and cost-effective voltammetric method for the quantification of SA in bovine milk.

## 2 Materials and Methods

### 2.1 Reagents and Solutions

All aqueous solutions used in this study were prepared from analytical-grade reagents and ASTM Type I ultrapure water (resistivity ≥18 MΩ cm). Supporting electrolyte solutions (0.1 to 1.0 mol L^–1^) were prepared with solid NaOH (Vetec, Rio de Janeiro, Brazil). A 0.2 mol L^–1^ SA stock solution was freshly prepared weekly by dissolving the appropriate mass of SA (ACS Científica, Sumaré, Brazil) in 2.5 mol L^–1^ NaOH. This stock solution was stored in a refrigerator and used daily to prepare more diluted SA solutions (10 to 500 μmol L^–1^), required for voltammetric characterization and calibration. An aqueous solution containing 15% (m/v) of ZnSO_4_ (ACS Científica, Sumaré, Brazil) was used for milk sample pretreatment.

### 2.2 Instrumentation and Apparatus

Ultrapure water used in this study was obtained from a Gehaka MS3000 purification system (Gehaka, São Paulo, Brazil). Electrochemical experiments were performed using a PGSTAT101 potentiostat/galvanostat (Metrohm-Autolab, Utrecht, Netherlands) interfaced with a personal microcomputer and controlled with the software NOVA 2.1.6. The reference electrode was a miniaturized lab-made Ag/AgCl/KCl-saturated electrode,^43^ and the auxiliary electrode was a spiral Pt wire. The surface morphology of Cu electrodes was examined using a scanning electron microscope TESCAN Vega 3 (Brunn, Czech Republic) operated at an electron beam energy of 20 kV and controlled via Vega TC software. Scanning electron microscopy (SEM) images were acquired for both freshly polished and electrochemically conditioned electrodes to compare their surface features and evaluate the effects of electrochemical modification. For the pretreatment of milk samples, a microcentrifuge (model DT-10K–BI, Daiki, Osaka, Japan) and an ultrasonic bath (model LS-3DA-3/X, Ultronique, Indaiatuba, Brazil) were employed.

### 2.3 Fabrication of the Copper Working Electrode

The copper working electrode employed in this study was designed based on two miniaturized electrochemical cells previously developed by our research group.^42,44^ To align with sustainable practices, the electrode was constructed using repurposed materials: a reused 1 mL Combitip (Eppendorf, Hamburg, Germany) was the electrode holder, while a copper wire (Ø = 1.3 mm) commonly used in domestic electrical installations, formed the working electrode surface. This approach significantly reduced material costs to less than 2 US dollars per device. The electrode was fabricated by sealing the copper wire into the Combitip using epoxy glue (Araldite, Tek Bond, Embu das Artes, Brazil) and curing the assembly at room temperature (24 ± 1 °C) for 6 h. The bottom portion of the cured electrode was sequentially sanded with 220 and 2500-grit sandpapers to remove excess epoxy glue and produce a smooth copper surface.

Prior to voltammetric experiments, the copper surface was polished with a 0.5 μm alumina suspension (Teclago, Vargem Grande Paulista, Brazil) on a polishing cloth. Representative images of the components and the assembled copper electrode are presented in Figure S1 of the Supporting Information. Figure 1 displays the assembled copper electrode (Figure 1a) and its freshly polished surface (Figure 1b).

**Figure 1:**
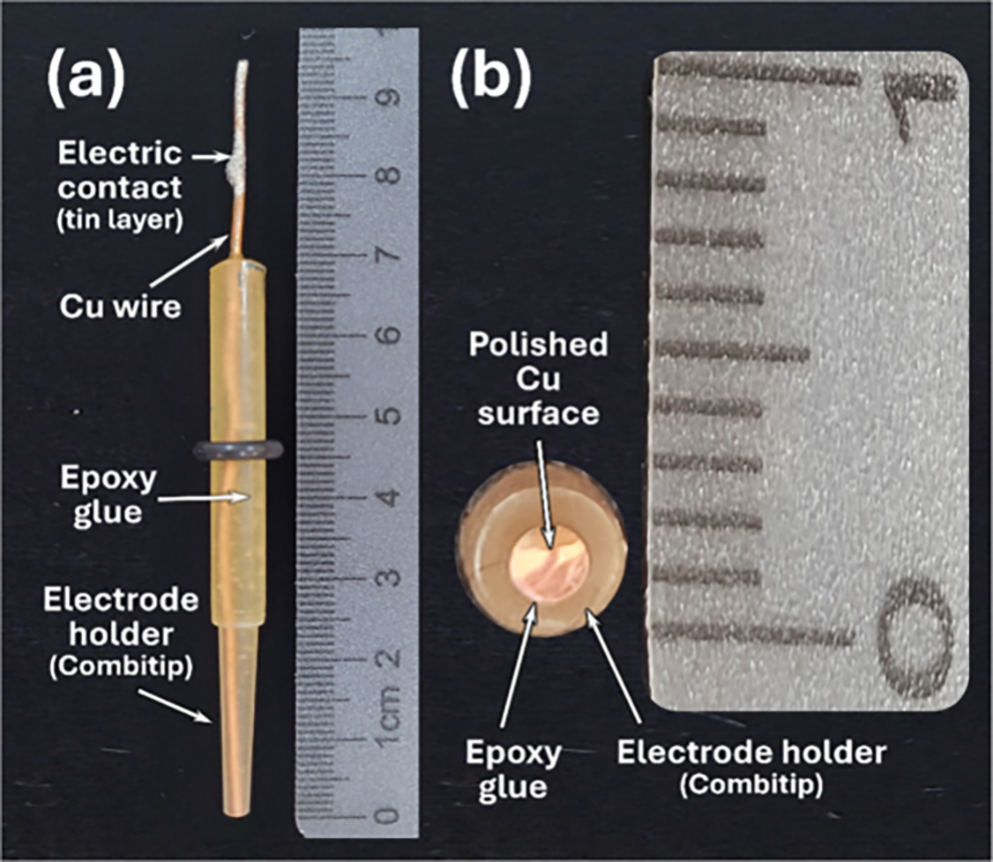
Images of the assembled copper electrode (a) and its polished surface (b).

Before starting each study, the copper working electrode was electrochemically conditioned through ten potential cycles (0 to +0.75 V vs. Ag/AgCl/KCl_sat_ at 100 mV s^–1^) in a 0.1 mol L^–1^ NaOH solution. The same conditioned electrode was then used for all subsequent measurements within that study. Electrode reconditioning was only performed when starting a new set of experiments or when the electrode was removed from the solution.

### 2.4 Milk Samples Pretreatment

Milk samples were stored in a refrigerator until analysis. A 500 μL aliquot of refrigerated milk was transferred to a 1.5 mL polypropylene microcentrifuge tube, followed by the sequential addition of 100 μL of 15% (m/v) ZnSO_4_ aqueous solution and 400 μL of 2.5 mol L^–1^ NaOH solution. The mixture was placed in an ultrasonic bath for 10 min and then centrifuged at 12,000 rpm for 5 min. After centrifugation, a solid phase formed and floated to the top of the solution. A 500 μL aliquot of the clear liquid beneath the solid phase was carefully collected using a micropipette and transferred to the electrochemical containing 4.5 mL of ultrapure water. This sample pretreatment combines the well-known protein precipitation capability of zinc sulfate^45^ and the conversion of SA to salicylate ion in alkaline media. The ionization of SA releases it from noncovalent interactions with proteins or fatty material in the milk matrix.^11^ For addition-recovery experiments, 5 μL of ZnSO_4_ solution was replaced with 5 μL of an SA stock solution at the desired concentration.

## 3 Results and Discussion

### 3.1 Characterization of the Cu Wire Electrode

Figure 2a displays the cyclic voltammogram of a freshly polished Cu wire electrode in 0.1 mol L^–1^ NaOH, recorded from −1.0 to +0.65 V. The voltammetric profile is consistent with the behavior of Cu electrodes in alkaline solutions.^37,46,47^ During the forward scan, three anodic peaks, denoted A (−0.331 V), B_1_ (−0.087 V), and B_2_ (+0.162 V) are observed. Peak A is widely attributed to the formation of cuprous oxide (Cu_2_O), as described by eq 1

1 $$2\mathrm{Cu}+2OH^{-}⇌{\mathrm{Cu}}_{2}O+H_{2}O+2e^{-}$$

**Figure 2:**
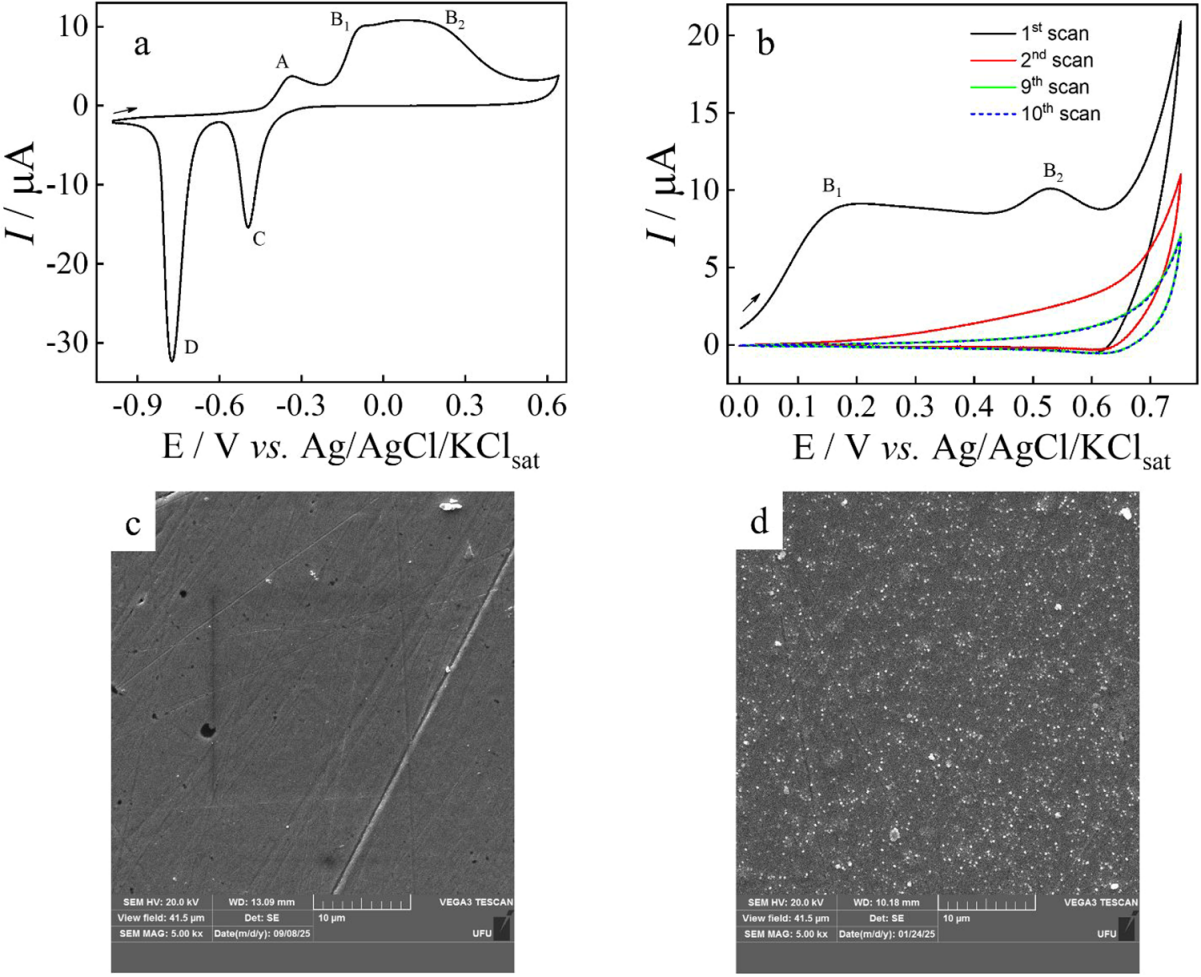
(a) Cyclic voltammogram of a freshly polished Cu wire electrode (Ø = 1.3 mm) in 0.10 mol L^–1^ NaOH at 50 mV s^–1^. (b) Successive cyclic voltammograms of the Cu wire electrode recorded from 0 to +0.75 V at 100 mV s^–1^ in 0.1 mol L^–1^ NaOH at 100 mV s^–1^. (c) SEM image of the freshly polished Cu wire electrode. (d) SEM image of the Cu wire electrode after 10 potential cycling as in (b).

The attribution of peaks B_1_ and B_2_ remains complex and debated, with two principal reaction pathways described in the literature. One pathway ascribes these peaks to the formation of copper hydroxide species—either soluble Cu­(OH)_4_^2–^ or insoluble Cu­(OH)_2_—through the oxidation of metallic Cu or Cu_2_O. An alternative pathway links these peaks to the formation of cupric oxide (CuO), either by direct oxidation of Cu or by dehydration of Cu­(OH)_2_.^37,46,48^ Regardless of the mechanism, the predominant species formed on the electrode surface at peaks B_1_ and B_2_ are Cu­(OH)_2_ and CuO. The electrochemical steps leading to their formation from metallic copper are represented by eqs 2 and 3

2 $$\mathrm{Cu}+2OH^{-}⇌\mathrm{Cu}(\mathrm{OH}{)}_{2}+{2e}^{-}$$

3 $$\mathrm{Cu}+2OH^{-}⇌\mathrm{CuO}+H_{2}O+{2e}^{-}$$

It is important to note that these equations represent simplified models. Both CuO and Cu­(OH)_2_ may also arise from the electrooxidation of Cu_2_O, and various soluble or insoluble copper hydroxide species may form depending on hydroxide ion concentration, Cu^2+^ concentration, and scan rate.^49^ Despite these uncertainties, Giri and Sarkar,^48^ using X-ray photoelectron spectroscopy (XPS) found evidence that peak B_1_ is predominantly associated with Cu­(OH)_2_ formation, whereas peak B_2_ corresponds mainly to CuO formation.

During the reverse scan, two cathodic peaks C (−0.494 V) and D (−0.775 V), are observed and have been attributed, respectively, to the reduction of CuO to Cu and Cu­(OH)_2_ or Cu­(OH)_4_^2–^ to Cu.^48^ Alternatively, some authors assign peak C to the reduction of Cu_2_O to Cu and peak D to the reduction of CuO/Cu­(OH)_2_ to Cu.^50^ To distinguish between these assignments, cyclic voltammograms were recorded with the switch potential set immediately after peak A (Figure S2), revealing a new cathodic peak, A′, between peaks C and D. The appearance of A′ indicates that peaks C and D should not be ascribed to Cu_2_O reduction, consistent with Giri and Sakar’s findings.^48^ These experiments demonstrate that the cathodic behavior of Cu electrodes is a complex multistep process whose mechanism, like that of anodic reactions, remains debated in the literature.

Peak A in Figure 2a is associated with the formation of a Cu_2_O monolayer on the copper surface, and the corresponding voltammetric charge can be used to estimate the electrochemically active surface area (ECSA). The charge density required to form a Cu_2_O monolayer is reported to be approximately 360 μC cm^–2^.^48,51^ For peak A, a charge of 10.3 ± 0.5 μC (*n* = 3) was obtained, yielding an ECSA of 0.029 ± 0.001 cm^2^. Given the geometric area of the copper wire electrode (0.013 cm^2^), the resulting roughness factor—defined as the ratio of real to geometric surface area—was 2.2 ± 0.1. This value exceeds the roughness factor of 1.6 reported by Giri and Sarkar,^48^ which is consistent with the extensive sanding used to remove excess epoxy glue (Section 2.3). Such mechanical treatment likely increased surface irregularities, thereby enhancing the roughness factor and potentially improving the electrode’s performance in electroanalytical applications.

When the potential window was restricted from 0 to +0.75 V, we observed a better definition of peak B_2_ during the first scan (Figure 2b). However, the intensities of peaks B_1_ and B_2_ progressively decreased over successive potential cycling, reaching a stable voltammetric profile after 10 cycles. This decrease has been reported in literature and is ascribed to the irreversible electrochemical growth of a CuO/Cu­(OH)_2_ layer, which passivates the copper surface and inhibits further oxidation.^37,40^ SEM images were used to investigate the formation of this layer. Comparative images of a freshly polished Cu wire electrode and one subjected to 10 potential cycles between 0 and +0.75 V were acquired (Figure 2c,d, respectively). The polished electrode (Figure 2c) exhibits a predominantly smooth surface, with some linear features resulting from polishing. In contrast, the electrochemically conditioned electrode (Figure 2d) shows a markedly rougher surface with dense, uniformly distributed microstructures, consistent with the electrochemical growth of a covering layer. Literature reports,^49^ indicate that anodizing Cu foils at low current densities in 0.1 mol L^–1^ KOH favors the formation of CuO over Cu­(OH)_2_, whereas higher current densities and KOH concentrations as high as 6.0 mol L^–1^ favor Cu­(OH)_2_ formation. Thus, using the electrochemical protocol of Figure 2c, the resulting covering layer likely consists mainly of CuO. Energy-dispersive X-ray spectroscopy (EDS) confirmed Cu and O as the main elements on both surfaces, but was unable to resolve differences in O content between the electrodes, for which the oxygen weight percentage ranged from 3 to 6%.

Open-circuit potential (OCP) measurements were recorded for 1800 s on both freshly polished and electrochemically conditioned Cu wire electrodes (Figure S3). These measurements provide valuable insight into the thermodynamic state of the electrode–electrolyte interface, reflecting the presence and relative stability of redox-active surface species. For the freshly polished electrode, a stable OCP of −0.375 V vs. Ag/AgCl/KCl_sat_ (−0.178 V vs. SHE) was achieved after an initial 300 s transient period. This OCP value falls within the Cu_2_O stability domain of the copper Pourbaix diagram at pH 13 (corresponding to 0.1 mol L^–1^ NaOH).^52^ This result suggests that a freshly polished surface plausibly becomes covered by a thin Cu_2_O layer that forms spontaneously upon exposure to the alkaline electrolyte. In contrast, the conditioned electrode exhibited a significantly more positive OCP of −0.164 V vs. Ag/AgCl/KCl_sat_ (+0.033 V vs, SHE), which lies within the CuO stability domain.^52^ The observed differences in OCP for the freshy polished and conditioned electrode provide strong evidence that the conditioning step promotes the formation of a more oxidized, CuO-rich surface, stable under open-circuit conditions. Such a surface is presumably responsible for the enhanced antifouling properties and catalytic activity toward SA electrooxidation described in the next section. It should be noted that the potential drift observed during the first 300 s for both electrodes is not indicative of surface instability but rather reflects typical surface reorganization or equilibration following immersion or transition from a polarized to an open-circuit state.

The thickness of the CuO-rich layer was estimated using the approach described by Kunze et al.^53^ For this estimation, the layer was assumed to be composed entirely of CuO, which is reduced to metallic Cu at peak C in Figure 2a. Under this assumption, the voltammetric charge (*Q*) associated with peak C was used to calculate the surface coverage (Γ) according to eq 4

4 $$\Gamma =\frac{Q}{\mathrm{nFA}}$$

Where *n* is the number of exchanged electrons (2), *F* is the Faraday constant (95,485 C mol^–1^), and *A* is the electrode area (0.029 cm^2^). An average charge of 18 ± 1 μC (*n* = 3) was obtained for peak C, yielding a surface coverage of (3.2 ± 0.2) × 10^–9^ mol cm^–2^. The thickness of the CuO layer (δ) was then estimated using eq 5

5 $$\delta =\frac{M_{\mathrm{CuO}}\times \Gamma }{\rho_{\mathrm{CuO}}}$$

Where *M*_CuO_ is the molar mass of CuO (79.55 g mol^–1^) and ρ_CuO_ its density (6.42 g cm^–3^).^53^ The calculated thickness was 3.9 ± 0.2 nm, in good agreement with reported values for CuO/Cu­(OH)_2_ films electrochemically grown in 0.1 mol L^–1^ NaOH (2–4 nm).^53^ Despite this agreement, this result should be regarded as a rough estimate, since even under conditions favoring CuO formation, the film invariably contains some Cu­(OH)_2_.^49^

### 3.2 Voltammetric Behavior of SA at the Cu Wire Electrode

Figure 3a shows that a freshly polished copper wire electrode is not suitable for the voltammetric detection of SA, producing a poorly defined anodic peak that overlaps with the CuO formation peak (B_2_). Moreover, the SA signal was unstable, with its intensity and shape varying across successive scans. To address this issue, the copper wire electrode was conditioned by performing 10 potential cycles between 0 and +0.75 V at 100 mV s^–1^ in 0.1 mol L^–1^ NaOH, aiming to form the CuO-rich layer on the electrode surface. A scan rate of 100 mV s^–1^ was selected for the electrode conditioning, as it offers an optimal balance between efficient surface modification and practical experimental duration. As shown in Figure 3b, this conditioning resulted in a markedly cleaner background voltammogram (black curve), which is essential for electroanalytical applications. This conditioning also enhanced the voltammetric response for SA, yielding an intense and well-defined anodic peak at +0.52 V (red curve).

**Figure 3:**
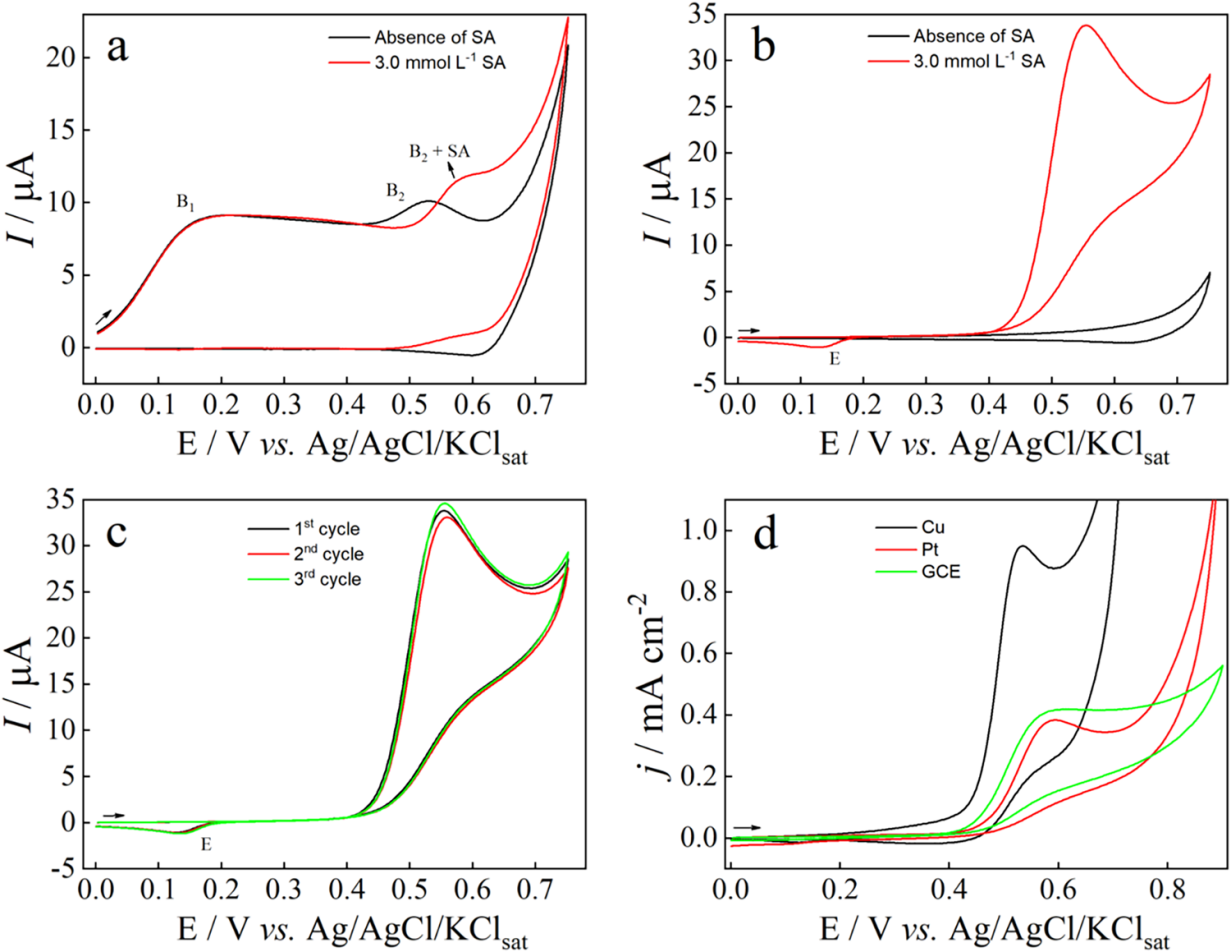
(a) Cyclic voltammograms of a freshly polished Cu wire electrode (Ø = 1.3 mm) in 0.10 mol L^–1^ NaOH at 100 mV s^–1^ recorded in the absence and presence of 3.0 mmol L^–1^ SA. (b) Cyclic voltammograms of the Cu wire electrode preconditioned by 10 potential cycles between 0 and +0.75 V at 100 mV s^–1^ in 0.1 mol L^–1^ NaOH, recorded in the absence and presence of 3.0 mmol L^–1^ SA. (c) Cyclic voltammograms recorded in the presence of 3.0 mmol L^–1^ SA under the same conditions as in (b), with a new electrochemical conditioning performed before each cycle. (d) Cyclic voltammograms recorded in 0.10 mol L^–1^ NaOH containing 3.0 mmol L^–1^ SA at 100 mV s^–1^ using different working electrodes. (Black Solid Dash) Cu (Ø = 1.3 mm), (Red Solid Dash) Pt (Ø = 3.0 mm), and (Green Solid Dash) GCE (Ø = 3.0 mm).

The CuO-rich layer is widely recognized for its high electrocatalytic activity toward the oxidation of various organic compounds, mainly glucose, although the mechanism explaining this activity remains debated. Proposed explanations include mediation by Cu­(III) species or catalytic effects arising from hydroxyl adsorption, combined with the semiconductive properties of the CuO layer.^52^ Detailed discussions on glucose electrooxidation at Cu electrodes are provided by Aun et al.^34^ Regardless of the exact mechanism, Figure 3b demonstrates that the CuO-rich layer also facilitates the electrooxidation of SA, markedly enhancing its voltammetric signal. Electrode conditioning not only enhanced the voltammetric response of SA but also ensured a highly repetitive voltammetric profile as shown in Figure 3c. For consecutive voltammetric scans using the same electrode after independent conditioning, an RSD of 2.0% (*n* = 3) was obtained for *i*_pa_ of 3.0 mmol L^–1^ SA (Figure 3c). When the electrode was conditioned only once and the solution was stirred between consecutive scans, the RSD decreased to 0.5% (*n* = 5), indicating negligible short-term electrode fouling and demonstrating that the modified surface is suitable for multiple scans (Figure S4). Therefore, for SA electrooxidation, the CuO-rich layer plays a bifunctional role, providing both an enhanced electron transfer rate and antifouling protection, functionalities previously reported in literature.^37^ As shown in Figure 3d, compared to glassy carbon electrode (GCE) and Pt, the Cu wire electrode exhibited the highest peak current density and the lowest peak potential for SA electrooxidation, highlighting it as a cost-effective and high-performance platform for SA sensing.

According to the literature, the anodic peak at +0.52 V (Figure 3b, red curve) corresponds to the oxidation of SA to its phenoxyl radical through the loss of one-electron and one proton.^9,26^ The phenoxyl radical can undergo different pathways, including further oxidation to soluble carboxylic acids, coupling to form quinone derivatives, and subsequent polymerization resulting in passivating polymeric films.^9,26^ Figure S5 illustrates a simplified reaction pathway for the electrooxidation of salicylic acid. The absence of the corresponding cathodic peak in the reverse scan has been reported by several authors and attributed either to an irreversible electron transfer^26,28,54^ or to a rapid chemical step coupled to electron transfer (EC_i_ mechanism).^24,25,55^ As shown in Figure S4, no electrode fouling was observed during consecutive voltammetric scans, indicating that under these experimental conditions, no passivating polymeric film is formed.

The cyclic voltammograms in Figure S6 reveal the appearance of a new redox couple, *E*/*E*′, in the second voltammetric scan, with a formal potential of +0.16 V. Its appearance only in the second scan indicates that it arises from a product generated during the initial electrooxidation of SA at +0.52 V. Similar voltammetric behavior has been observed for SA on carbon electrodes, with this redox couple attributed to the quinone moiety of SA electrooxidation products, such as carboxyl-ortobenzoquinone derivatives.^24–26,55^ The changes in the voltammetric profile observed in the second scan, relative to the first (Figure S6), cannot be attributed to electrode fouling, as the original response is fully restored simply by stirring the solution.

To further investigate the voltammetric behavior of SA on the preconditioned Cu wire electrodes, cyclic voltammograms were recorded at increasing scan rates with stirring between scans. As shown in Figure S7a, the peak potential of SA shifts positively with increasing scan rates, indicating an irreversible electron transfer process, consistent with previous reports.^24–26,55^ Analysis of the log *i*_pa_ vs. log v plot (Figure S7b) revealed a linear relationship: log *i*_p_ (μA) = 0.51 + 0.44 log v (mV s^–1^), *R* ^2^ = 0.99935. The slope of 0.44 ± 0.03 (*n* = 3) is statistically equivalent to 0.5 at the 95% confidence level, confirming that SA electrooxidation on Cu electrodes is a diffusion-controlled process.

### 3.3 Analytical Performance of DPV and SWV for SA Determination

The analytical performance of differential pulse voltammetry (DPV) and square wave voltammetry (SWV) for SA determination was compared. The supporting electrolyte was 0.1 mol L^–1^ NaOH and the operational parameters of each technique were optimized (Table S1). Before each study, the Cu wire electrode was electrochemically conditioned by recording 10 potential cycles between 0 and +0.75 V at 100 mV s^–1^ in 0.1 mol L^–1^ NaOH, to form the CuO-rich layer on the electrode surface. The SA concentration used for DPV optimization was 50 μmol L^–1^ and for SWV 100 μmol L^–1^. This concentration changing was necessary since SWV, at the initial conditions, could not detect SA concentrations below 100 μmol L^–1^.

The baseline corrected DPV and SWV voltammograms recorded before and after optimizing the voltammetric parameters are shown in Figure S8. The baselines were corrected using the moving average mode with window size of 2.0. As illustrated in Figure S8, optimizing the voltammetric parameters enhanced the voltammetric response of SA for both DPV and SWV. Figure 4 shows the DPV and SWV voltammograms recorded at increasing SA concentrations and the corresponding analytical curves.

**Figure 4:**
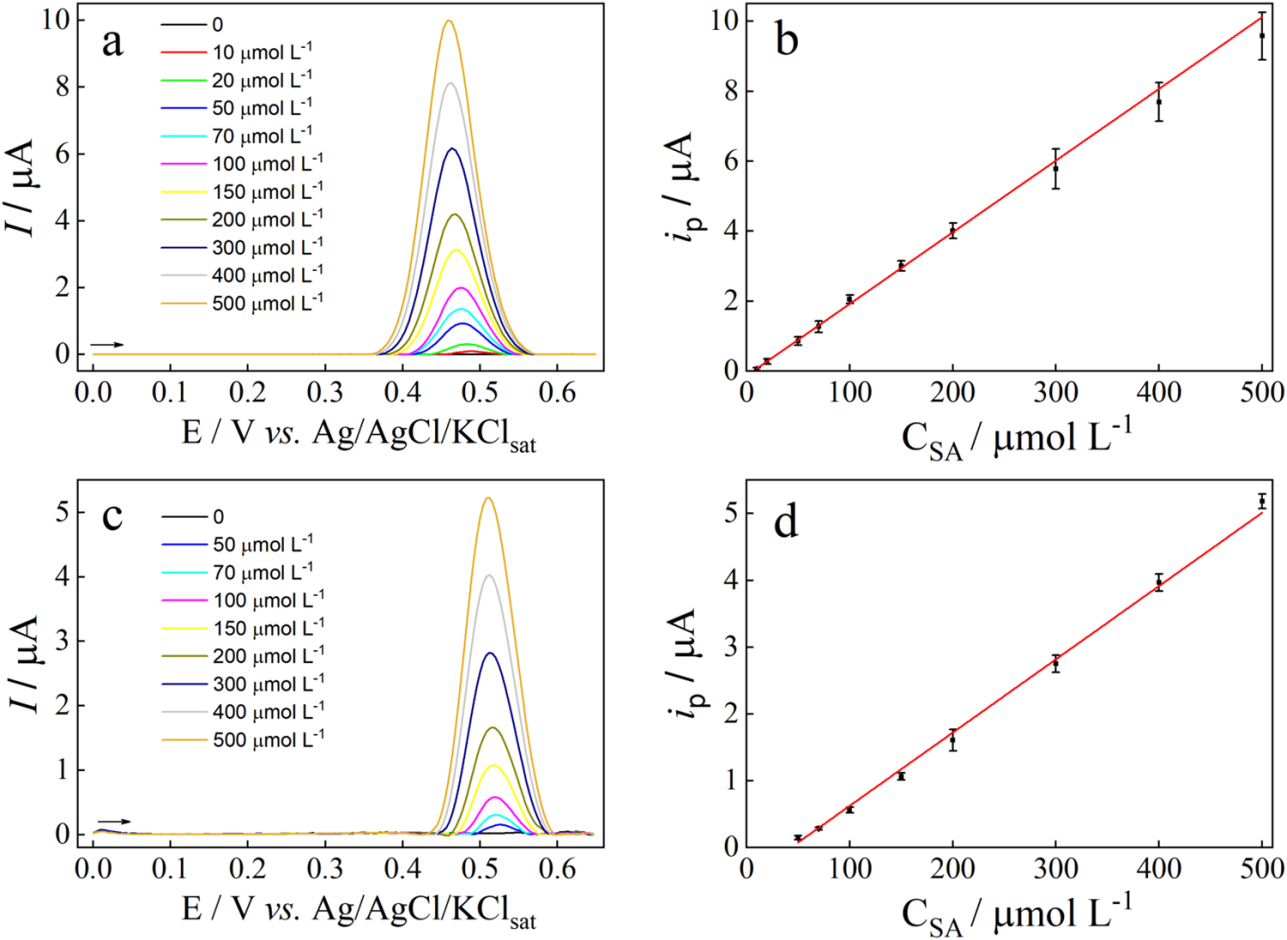
Baseline-corrected differential pulse (a) and square wave (c) voltammograms recorded in 0.1 mol L^–1^ NaOH at increasing SA concentrations. Corresponding analytical curves for SA using DPV (b) and SWV (d) Voltammetric conditions for DPV: Δ*E* = 50 mV; pulse width = 25 ms, and step potential = 5 mV. Voltammetric conditions for SWV: Δ*E* = 25 mV; *f* = 50 Hz, and Δ*E*_s_ = 4 mV. The baselines were corrected using the moving average mode with window size of 2. Error bars represent the standard deviation of *i*_pa_ (*n* = 3).

A cathodic shift in the voltammetric peaks was observed at increasing SA concentrations with both DPV and SWV (Figure 4a,c); however, this effect was identified as an artifact introduced by baseline correction, since no such shift was present in the raw voltammograms. Between successive SA additions, the solution was stirred to ensure proper mixing of the added SA, but no electrode reconditioning was performed. The analytical curve with DPV was linear from 10 to 500 μmol L^–1^, according to the equation: *i*_p_ (μA) = −0.129 + 0.0205*C*_SA_ (μmol L^–1^), *R* ^2^ = 0.99767. LOD and LOQ were calculated from the equations: $\mathrm{LOD}=\frac{3\times {\mathrm{sd}}_{B}}{S}$ and $\mathrm{LOQ}=\frac{10\times {\mathrm{sd}}_{B}}{S}$ , where *S* is the slope of the analytical curve and sd_B_ is the standard deviation for the blank signal. The sd_B_ was estimated from the standard deviation of the intercept from the analytical curve. The LOD and LOQ achieved with DPV were 3 and 10 μmol L^–1^, respectively. Using SWV, a linear range from 50 to 500 μmol L^–1^ was observed, with the equation: *i*_p_ (μA) = −0.472 + 0.0109*C*_SA_ (μmol L^–1^), *R* ^2^ = 0.99515. LOD and LOQ were 10 and 33 μmol L^–1^, respectively. Therefore, DPV was selected for further experiments because it exhibited not only better sensitivity and a lower LOD but also produced a more intense analytical signal and a sharper, better-defined peak than SWV, enhancing both quantification accuracy and peak resolution.

Under the optimized conditions, analytical curves were constructed in triplicate using DPV both within a single workday and across three consecutive workdays (Figure S9). The slopes from these calibration curves were 0.0190 ± 0.0008 and 0.0208 ± 0.0009 μA μmol^–1^ L, respectively. Statistical analysis, using *t*- and *F*-tests, revealed that the calculated *t*- and *F*-values (2.59 and 1.27, respectively) were below their critical values (*t*_critical_ = 2.78 and *F*_critical_ = 19, for four degrees of freedom at a 95% confidence level).^56^ These results indicate no statistically significant differences between the slopes or their variances, confirming the method’s intermediate precision and demonstrating that calibration remains consistent whether conducted on a single day or over multiple workdays. Furthermore, *i*_pa_ for 100 μmol L^–1^ of SA was measured over eight workdays (Figure S10), yielding an RSD of 3.8%. Collectively, these findings demonstrate that the electrochemical conditioning protocol of the Cu wire electrode ensures repeatability both in the short and long-term. Table S2 compares the analytical performance of the Cu electrode with other electrochemical methods for SA determination. While not achieving the lowest LOD, the conditioned Cu wire electrode offers key advantages: cost-effectiveness (using a commercial copper wire), simplicity (no need for expensive chemicals or time-consuming modifications), excellent repeatability (via simple voltammetric conditioning), and easy fabrication with readily available materials. These attributes make it a practical and reliable alternative for SA sensing. Among the electrochemical methods listed in Table S2, only one has reported the voltammetric determination of SA in milk samples, employing a carbon paste electrode (CPE) modified with Ce/ZrO_2_.^27^ While CPEs are low-cost and easy to prepare, the synthesis of the Ce/ZrO_2_ modifier involved time- and energy-intensive procedures. Although the modified CPE achieved a slightly lower limit of detection (1.0 μmol L^–1^), the straightforward fabrication, low cost, and commercial availability of the Cu wire electrode make our method a more practical and competitive option for SA quantification in milk.

### 3.4 Selectivity Toward SA

An interference study was conducted to evaluate the selectivity of the Cu electrode for SA in the presence of substances commonly found in milk or added as adulterants. The tested interferents included saccharose (SAC), lactose (LAC), glycose (GLY), and galactose (GAL) sugars naturally present in bovine milk. Additionally, the effects of urea (URE) and uric acid (UA), which may be naturally present or fraudulently added to milk, were examined. Finally, the study assessed interference from substances illicitly used to extend milk shelf life, including citric acid (CIT), hydrogen peroxide (H_2_O_2_), sodium hypochlorite (HYP), and formaldehyde (FOR).

The interference study evaluated the effect of potential interferents on the voltammetric signal of 100 μmol L^–1^ SA. The average *i*_pa_ (*n* = 3) for SA alone was normalized to represent 100% signal, and its confidence interval (at the 95% confidence level) was calculated. Three voltammograms were then recorded for each SA-interferent mixture at molar rations of 1:0.5, 1:1, and 1:10. Interference was considered significant when *i*_pa_ deviated beyond the confidence interval of the SA-only signal. The results of the interference study are summarized in Figure 5.

**Figure 5:**
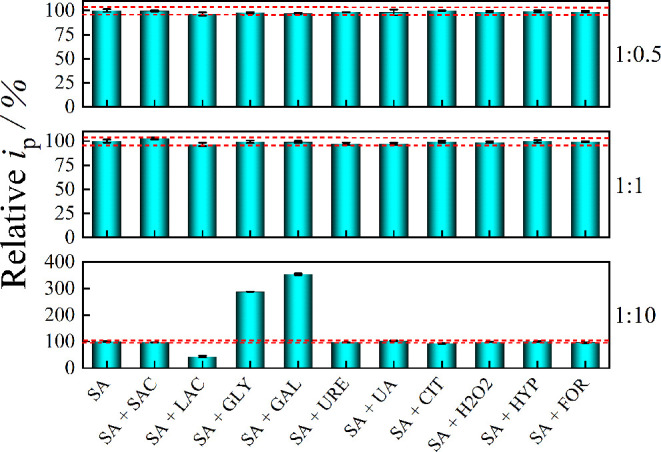
Influence of potential interfering species on the relative *i*_pa_ of 100 μmol L^–1^. The red dashed lines indicate the 95% confidence interval for the mean *i*_pa_ of SA alone (*n* = 3). Error bars represent the standard deviation of *i*_pa_ (*n* = 3) for each condition. The evaluated molar ratios of SA to interfering species are 1:0.5, 1:1, and 1:10.

The interference study indicated that none of the evaluated species interfered with SA determination when present at concentrations equal to or lower than that of SA. However, GLY and GAL showed significant interference at concentrations ten times higher than SA, increasing the SA signal by 300% and 400%, respectively. Lactose also interfered at a 10-fold higher concentration, decreasing the SA signal by 57%. The remaining evaluated species did not exhibit any significant interference, even at concentrations ten times higher than SA. These findings highlight the need to eliminate GLY and GAL during sample pretreatment, a procedure that will be detailed in the subsequent section. The interference from LAC can be effectively addressed using the standard addition calibration method. Therefore, despite the observed interferences, the developed method demonstrated satisfactory selectivity toward SA.

The differential pulse voltammograms obtained during the interference study are presented in Figure S11. Most of the tested species exhibited no electroactivity within the investigated potential window. However, GLY and GAL, at concentrations ten times higher than SA, generated voltammetric peaks that completely masked the SA signal. Additionally, H_2_O_2_ yielded a voltammetric peak at +0.05 V, while formaldehyde generated an anodic peak at +0.60 V at concentrations ten times higher than SA. Importantly, the voltammetric peaks for both H_2_O_2_ and FOR were well-separated from the SA peak. Consequently, the proposed method offers the capability of simultaneously determining SA, H_2_O_2_, and FOR. Alternatively, this method could be employed for qualitative screening of milk samples to detect these adulterants.

### 3.5 Milk Samples Pretreatment

Six distinct milk samples (skimmed, semiskimmed, whole lactose-free, whole, raw, and multi vitamin-fortified whole) were subjected to the sample pretreatment procedure detailed in the experimental section. This procedure offers several advantages, including low sample and reagent consumption (500 μL each), elimination of organic solvents, and short processing time, allowing for the preparation of eight samples in approximately 15 min. The pretreatment effectively separated the milk matrix into a distinct semisolid upper phase (containing protein and lipid fractions) and a consistently transparent lower phase (Figure S12), indicating effective removal of most matrix components.

It is important to emphasize that the pretreatment step involves adding 400 μL of 2.5 mol L^–1^ NaOH to 500 μL of the original milk sample. After dilution in the electrochemical cell, the resulting NaOH concentration is approximately 0.1 mol L^–1^, ensuring that, even during sample analysis, the solution pH remains sufficiently high to maintain the stability of the CuO-rich layer on the electrode surface. Another relevant aspect concerns the addition of Zn^2+^ ions, where 100 μL of a 15% (m/v) ZnSO_4_ solution is introduced into 500 μL of sample. This amount of Zn^2+^ largely exceeds the natural Zn^2+^ levels typically present in milk, rendering the contribution of endogenous Zn^2+^ negligible. Furthermore, Zn^2+^ is not electroactive within the investigated potential window and, therefore, should not interfere with the voltammetric signal of SA.

The environmental impact of our sample pretreatment was quantitatively assessed by using the AGREEprep metric,^57,58^ yielding a score of 0.54 (Figure 6). While indicating a moderate level of greenness with potential for optimization, this score represents a significant improvement over established methods including the 975.30 AOC method^10^ (0.27) and the method by Gentili et al.^11^ (0.17). This enhanced AGREEprep score reflects a reduced environmental footprint compared to these earlier methods, highlighting the progress toward more sustainable analytical practices in SA quantification.

**Figure 6:**
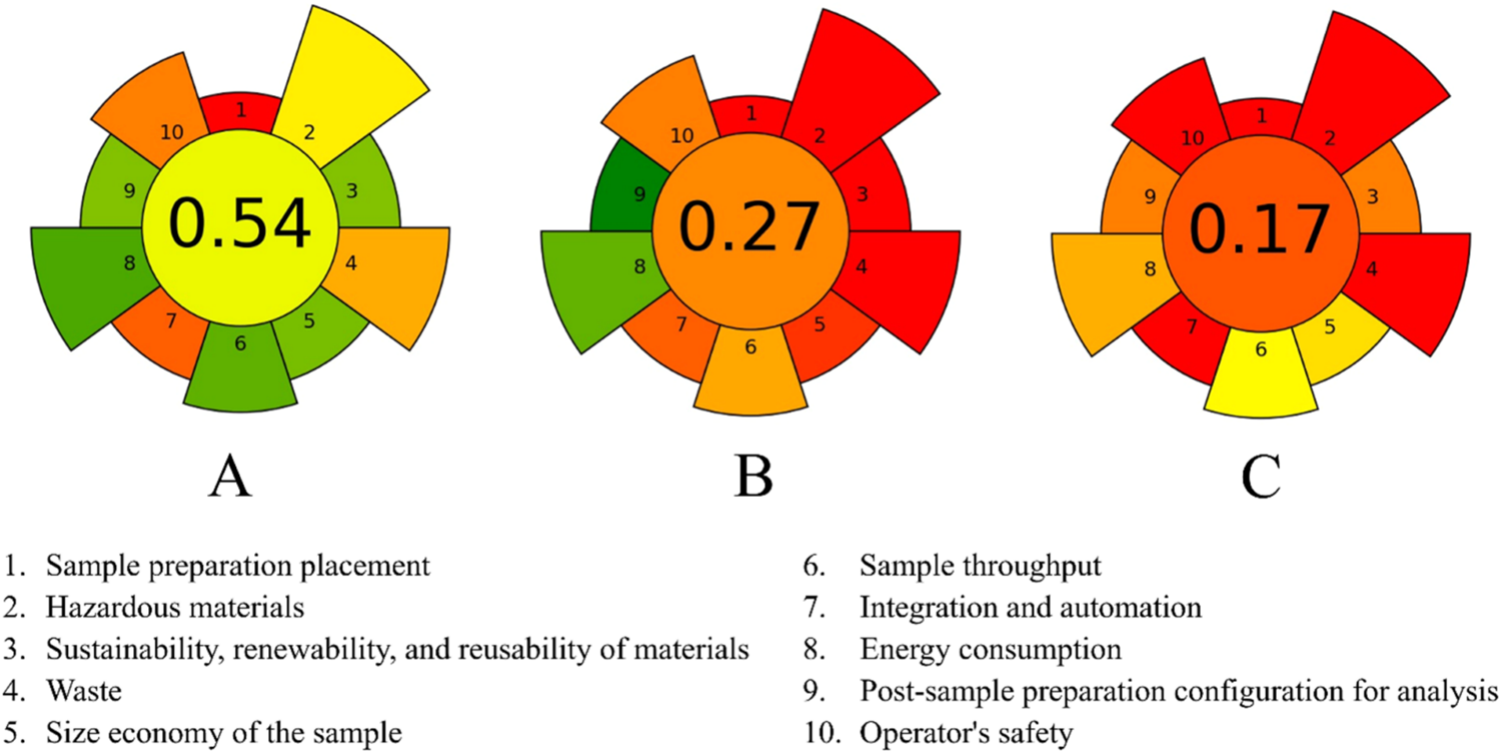
AGREEprep scores for procedures used in SA determination: (A) the proposed DPV method. (B) The 975.30 AOC method.^10^ (C) The method proposed by Gentili et al.^11^.

The proposed sample pretreatment includes two sequential dilution steps: an initial 1:1 dilution of the original sample, followed by a 1:10 dilution. In the second step, 500 μL of the intermediate extract is mixed with 4.5 mL of ultrapure water in the electrochemical cell, yielding a final NaOH concentration of 0.1 mol L^–1^, the optimized supporting electrolyte concentration. These sequential dilutions result in an overall dilution factor of 20, increasing the method’s effective LOD and LOQ to 60 μmol L^–1^ (8.2 mg L^–1^) and 200 μmol L^–1^ (27.6 mg L^–1^), respectively. Although these values are higher than those achieved by advanced techniques such as HPLC, they remain well below the typical SA concentrations reported in adulterated milk (400 to 500 mg L^–1^)^8^ confirming that the proposed method is suitable for quantifying SA in adulterated milk.

Differential pulse voltammograms were recorded over a range of SA concentrations in the final extracts from the six analyzed milk samples (Figure S13). The sample pretreatment effectively removed electroactive interfering species, including GAL and GLY, as evidenced by the absence of voltammetric peaks in the extracts prior to SA addition. Calibration curves constructed from these voltammograms are shown in Figure 7a, and the corresponding sensitivities (slopes of the calibration curves) obtained for each extract are shown in Figure 7b.

**Figure 7:**
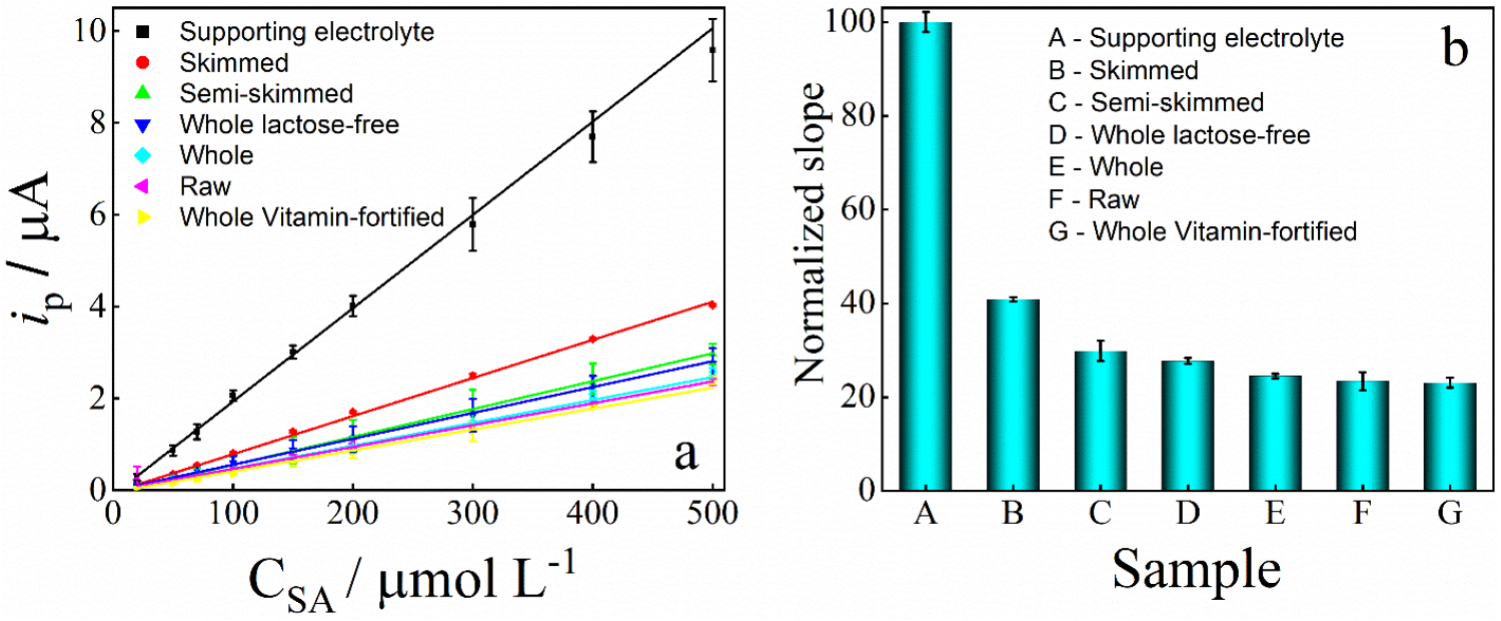
(a) Calibration curves obtained by DPV in the final milk extracts. (b) Normalized sensitivity obtained for each milk extract. Sensitivities were normalized relative to the sensitivity obtained in supporting electrolyte (defined as 100%).

Figure 7 shows that the sensitivity obtained in the milk extracts was lower than that obtained in the supporting electrolyte, indicating that the pretreatment did not fully eliminate matrix effects. Among the samples, the highest sensitivity was observed in the skimmed milk extract, suggesting a considerable influence of lipid content on SA sensitivity. As demonstrated in the selectivity study (Figure 5), LAC contributes to signal suppression; this is consistent with the lower sensitivity observed in the lactose-containing whole milk extract compared to the lactose-free whole milk extract (Figure 7b). In most samples, the observed matrix effects resulted in a sensitivity decrease exceeding 50% relative to the supporting electrolyte (Figure 7b). Consequently, to compensate for these varying matrix effects, the standard additions calibration method was employed for SA determination in spiked milk samples.

### 3.6 Sample Analysis

In all analyzed samples, no SA voltammetric peak was detected, indicating that it is either absent or present at concentrations below the LOD. Therefore, to evaluate the accuracy of the proposed method, addition-recovery experiments were performed using the six milk samples. Each sample was spiked with 138 mg L^–1^ of SA, corresponding to 50 μmol L^–1^ in the electrochemical cell. The spiked samples were then subjected to the proposed sample pretreatment procedure and SA concentrations were determined using standard additions calibration. Figure S14 shows the DPV voltammograms and the corresponding standard addition curves (insets).

As shown in Figure S14, the voltammetric profile and peak potentials for SA observed in the milk extracts remained consistent with those observed in the supporting electrolyte. Moreover, all standard addition curves exhibited satisfactory linearity, with *R* ^2^ values exceeding 0.99. The determined SA concentrations and recovery percentages for each analyzed sample are summarized in Table 1.

**Table 1:** Recovery Percentages and Determined SA Concentrations in Spiked Milk Samples

milk sample	added (mg L^–1^)^a^	found (mg L^–1^)^b^	recovery (%)	*t* ^c^
skimmed	138	146 ± 8	106 ± 6	1.7
semiskimmed	138	141 ± 3	102 ± 2	1.7
whole lactose-free	138	139 ± 4	101 ± 3	2.9
whole	138	148 ± 5	107 ± 3	3.4
raw	138	124 ± 5	91 ± 3	4.0
whole vitamin-fortified	138	141 ± 7	102 ± 5	0.7

a Added to the sample, resulting in a concentration of 50 μmol L^–1^ (6.9 mg L^–1^) in the electrochemical cell. Typical concentrations of SA used as a milk adulterant range from 400 to 500 mg L^–1^.^8^

b Average value ± standard deviation (*n* = 3).

c *t*_critical_ = 4.30 for 2 degrees of freedom and 95% confidence level.^56^

As shown in Table 1, recovery percentages ranged from 91 to 107%, with RSD values of 2.1–5.3%, meeting AOAC performance criteria for standard methods at 100 mg L^–1^ (expected recovery: 90–107%; RSD ≤5.3%).^59^ The *t* test confirmed no significant deviation of individual recoveries from 100% as all calculated *t*-values were below the critical value at the 95% confidence level. These results demonstrate the accuracy of the proposed voltammetric method for SA determination in milk samples.

It is important to highlight that all electrochemical measurements were conducted using a single copper electrode, which exhibited stable voltammetric responses throughout the experimental period. This sustained performance, combined with routine electrochemical conditioning at the beginning of each workday, demonstrates the electrode’s reusability and long-term stability.

## 4 Conclusions

This study demonstrated that a simple and cost-effective Cu electrode, fabricated from commercially available Cu wires, exhibited an enhanced voltammetric response for SA compared to Pt and GCE electrodes. This improvement is attributed to the reproducible formation of a CuO-rich layer on the electrode surface during a conditioning procedure using cyclic voltammetry in alkaline media. This electrocatalytic layer enhanced electron transfer kinetics for SA electrooxidation and prevented electrode fouling, ensuring stable voltammetric signals. Optimal analytical performance was achieved using DPV with a 0.1 mol L^–1^ NaOH solution as the supporting electrolyte. The method demonstrated satisfactory selectivity, as interference from GAL and GLY, observed at 10-fold excess concentrations, was effectively eliminated by the sample pretreatment. The interference from LAC was addressed using the standard addition method. Consequently, the proposed voltammetric method provided acceptable recoveries of SA in spiked milk samples.

The developed sample pretreatment offers some advantages, including low sample and reagent consumption, elimination of organic solvents, and short processing time. While its AGREEprep score (0.54) indicates moderate greenness, our approach demonstrates improved sustainability compared to previously reported methods. This pretreatment represents a promising tool for SA quantification and could be adapted for the analysis of other relevant analytes in milk.

## Supplementary Material



## Notes

Images of copper electrode components and assembly (Figure S1); cyclic voltammograms of the Cu wire electrode with different switch potentials (Figure S2); OCP measurements (Figure S3); additional voltammetric studies evaluating, signal stability (Figure S4), SA voltammetric behavior (Figure S6); scan rate (Figure S7), and parameter optimization (Figure S8); reaction pathway for SA electrooxidation (Figure S5), analytical curves obtained over multiple workdays (Figure S9); stability of SA signal (Figure S10); differential pulse voltammograms from interference studies (Figure S11); visual evidence of sample pretreatment efficacy showing matrix phase separation (Figure S12); voltammograms obtained from different milk matrices after extraction (Figure S13); standard-addition determinations of SA in spiked milk samples (Figure S14); optimization of voltammetric parameters (Table S1); and analytical performance of electroanalytical methods for SA determination reported in literature (Table S2) (PDF)
