# Supplementary material for: Cost-Effective Voltammetric Determination of Salicylic Acid in Milk Using Copper Wire Electrodes
Source: ACS Omega. 2025 Oct 10;10(41):48875–86. doi: 10.1021/acsomega.5c06973 (PMC12547540; doi:10.1021/acsomega.5c06973)
Supplement: Supplementary file 1 [file ao5c06973_si_001.pdf]

Supplementary material for

Cost-Effective Voltammetric Determination of Salicylic Acid in Milk Using Copper Wire Electrodes

Giulia C. P. Freitas[1], Vitoria B. Messias[2], Regina M. Takeuchi[1,2], André L. Santos[1,2]*

[1] Universidade Federal de Uberlândia, Instituto de Ciências Exatas e Naturais do Pontal, 38304-402, Ituiutaba, Brazil.

[2] Universidade Federal de Uberlândia, Instituto de Química, 38400-902, Uberlândia, Brazil.

* Corresponding author: alsantos@ufu.br

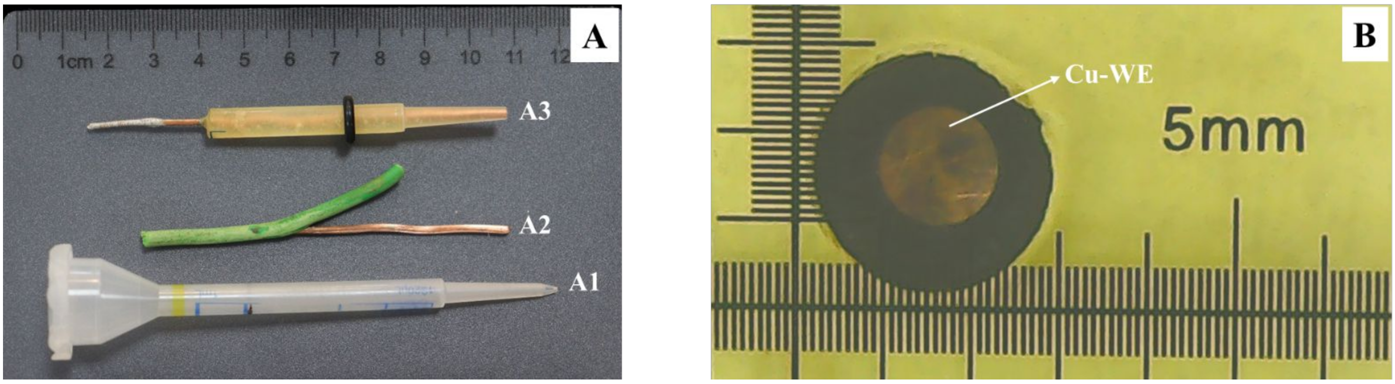

Figure S1. A) Images of the components used for fabricating the Cu working electrode (Cu-WE): (A1) reused Combitip, (A2) Cu wire, and (A3) assembled electrode. B) Image showing the surface of the Cu-WE.

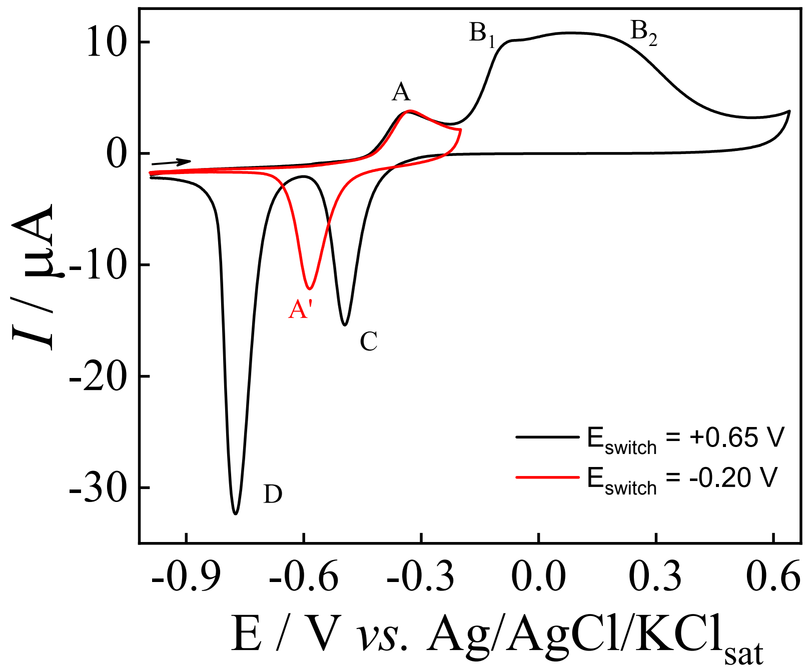

Figure S2. Cyclic voltammograms of a freshly polished Cu wire electrode (Ø = 1.3 mm) in 0.10 mol L^{-1} NaOH at 50 mV s^{-1}.

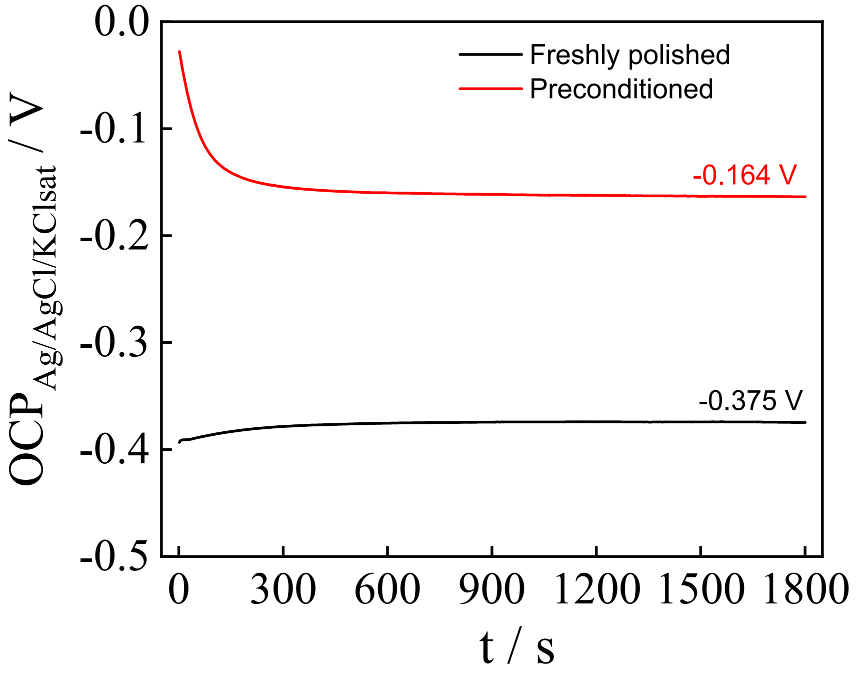

Figure S3. Open-circuit potential as a function of time for freshly polished and electrochemically conditioned Cu wire electrodes.

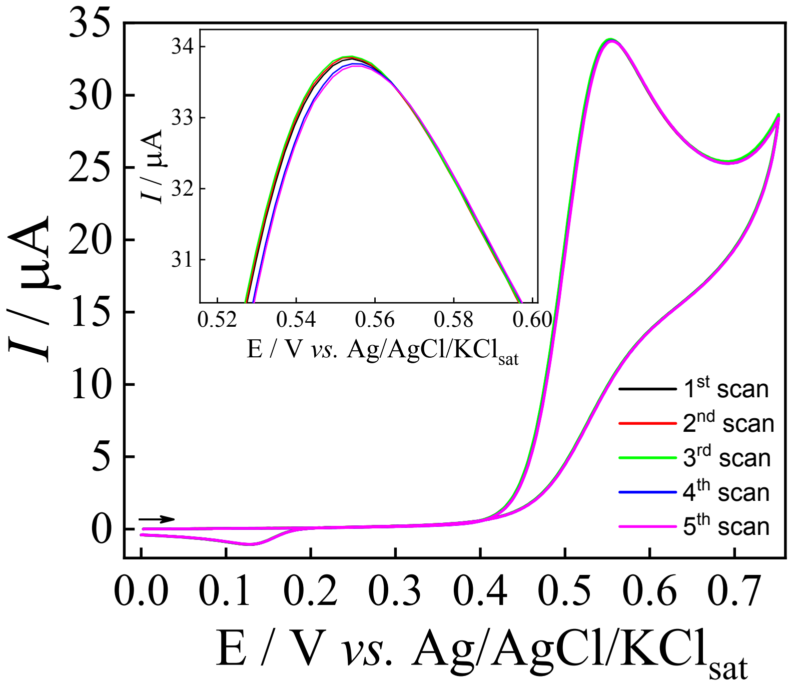

Figure S4. Five successive cyclic voltammograms recorded with a preconditioned Cu wire electrode (Ø = 1.3 mm) in 0.10 mol L^{-1} NaOH containing 3.0 mmol L^{-1} SA at 100 mV s^{-1}. The electrode was conditioned once, and the solution was stirred between scans without further reconditioning.

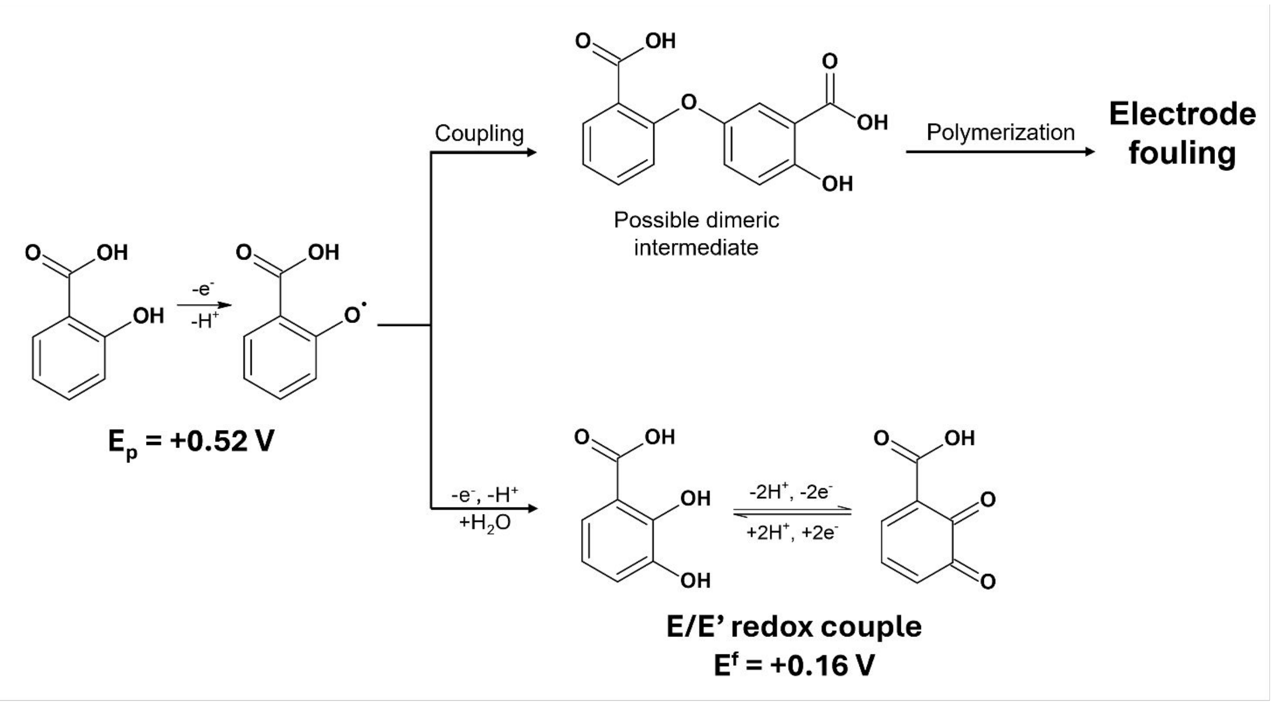

Figure S5. Simplified reaction pathway for the electrooxidation of salicylic acid

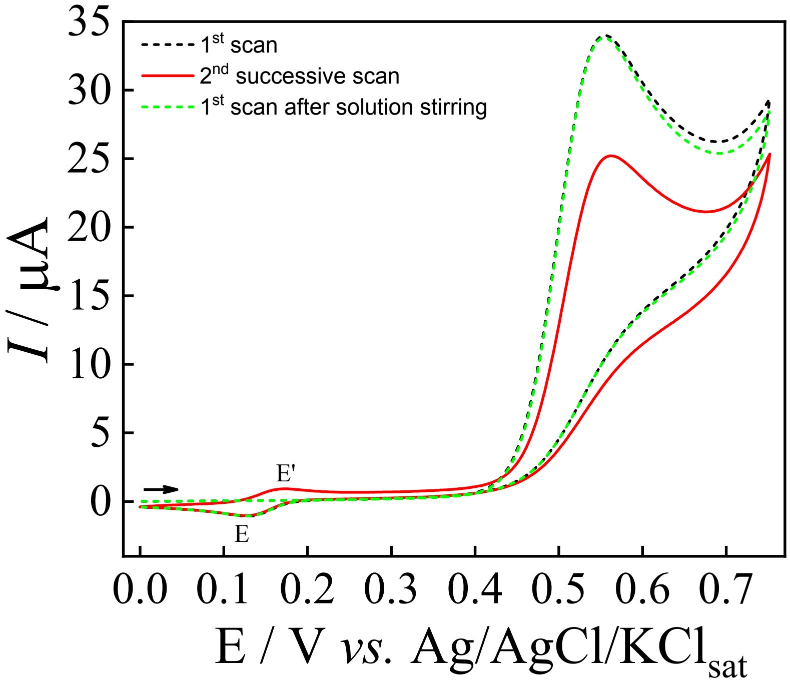

Figure S6. Cyclic voltammograms recorded with a preconditioned Cu wire electrode (Ø = 1.3 mm) in 0.10 mol L^{-1} NaOH containing 3.0 mmol L^{-1} SA at 100 mV s^{-1}. The black dashed and red solid lines correspond to successive scans, while the green dashed line was recorded after solution stirring.

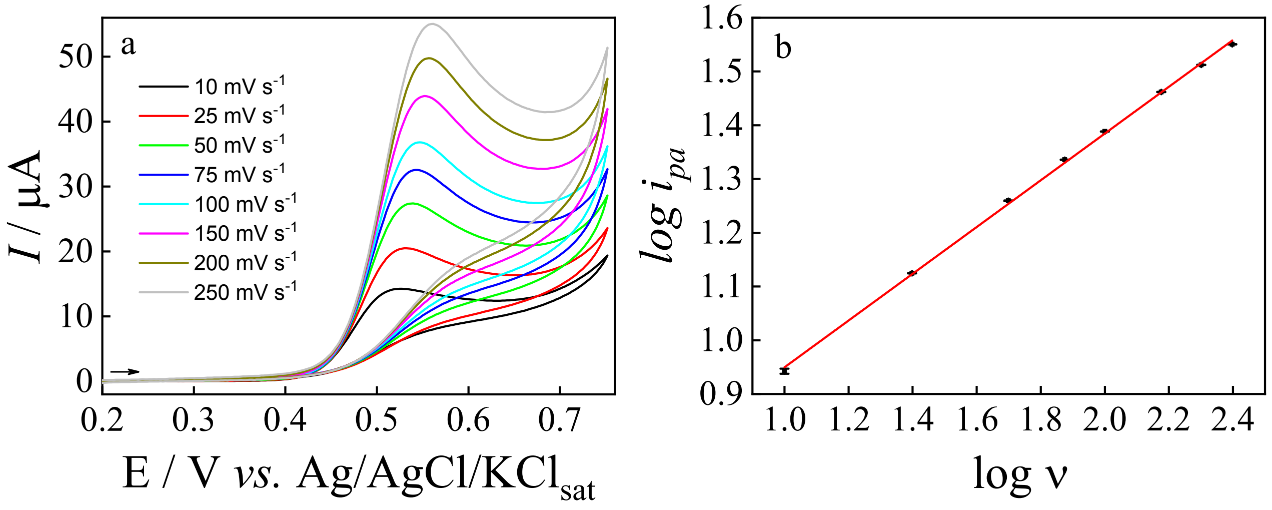

Figure S7. (a) Cyclic voltammograms recorded with a preconditioned Cu wire electrode (Ø = 1.3 mm) in 0.10 mol L^{-1} NaOH containing 3.0 mmol L^{-1} SA at different scan rates. (b) log i_{pa} vs. log v plot.

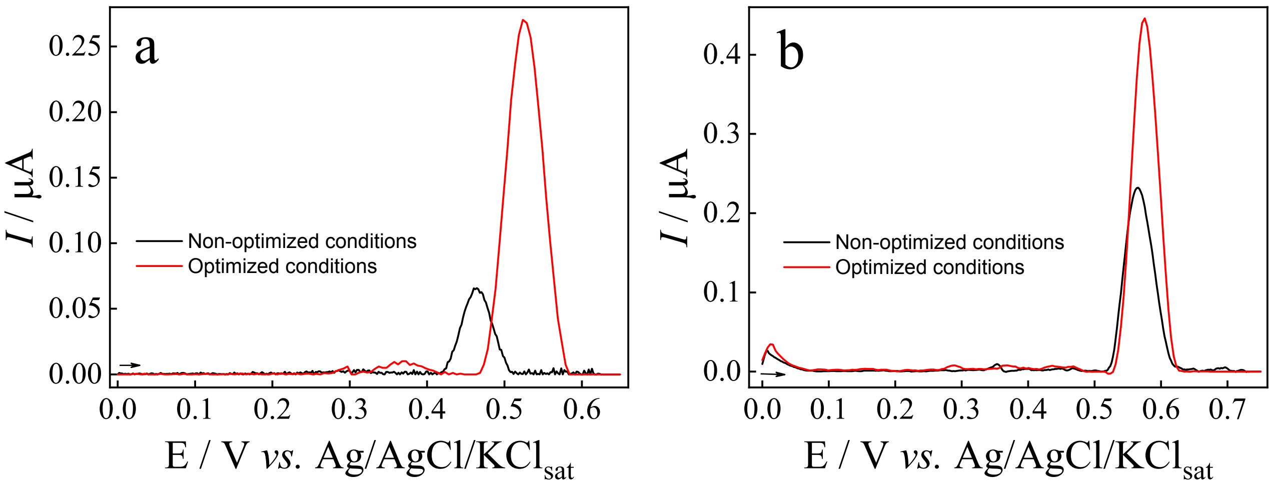

Figure S8. (a) Baseline corrected differential pulse voltammograms recorded with a preconditioned Cu wire electrode (Ø = 1.3 mm) in 0.1 mol L^{-1} NaOH containing 50 µmol L^{-1} SA at: (—) initial non-optimized conditions: pulse amplitude = 25 mV; pulse width = 50 ms; and step potential = 2 mV. (—) optimized conditions: pulse amplitude = 50 mV; pulse width = 25 ms; and step potential = 5 mV. (b) Baseline corrected square wave voltammograms recorded with a Cu electrode (Ø = 1.3 mm) in 0.1 mol L^{-1} NaOH containing 100 µmol L^{-1} SA at: (—) initial non-optimized conditions: pulse amplitude = 25 mV; frequency = 25 Hz; and step potential = 2 mV. (—) optimized conditions: pulse amplitude = 25 mV; frequency = 50 Hz; and step potential = 4 mV.

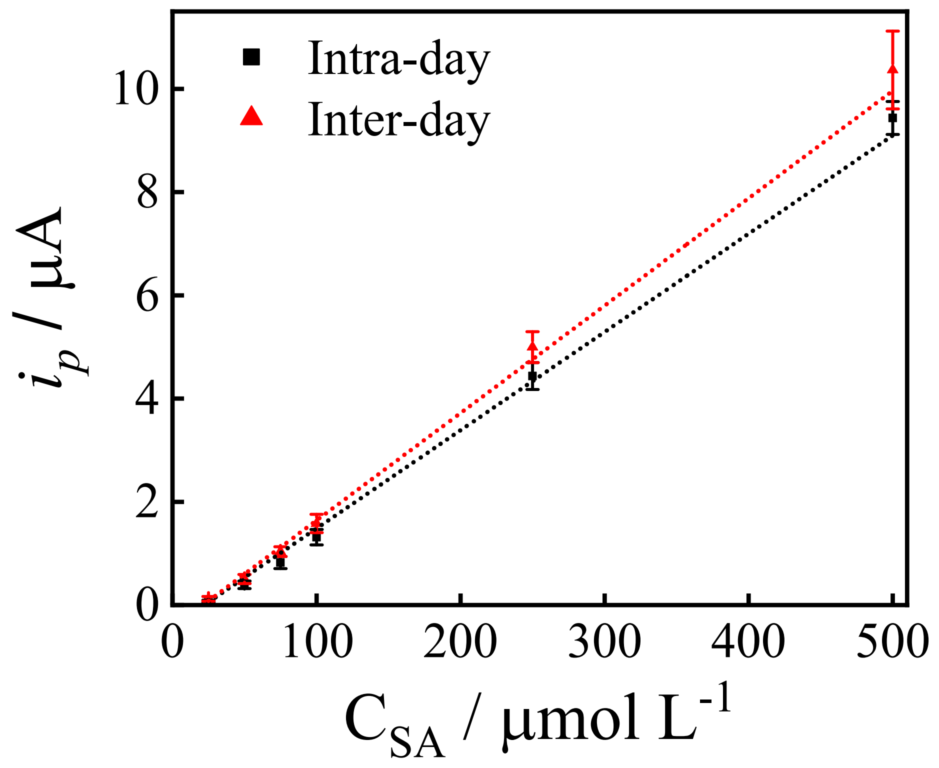

Figure S9. Inter- and Intra-day analytical curves for SA using DPV under optimized conditions.

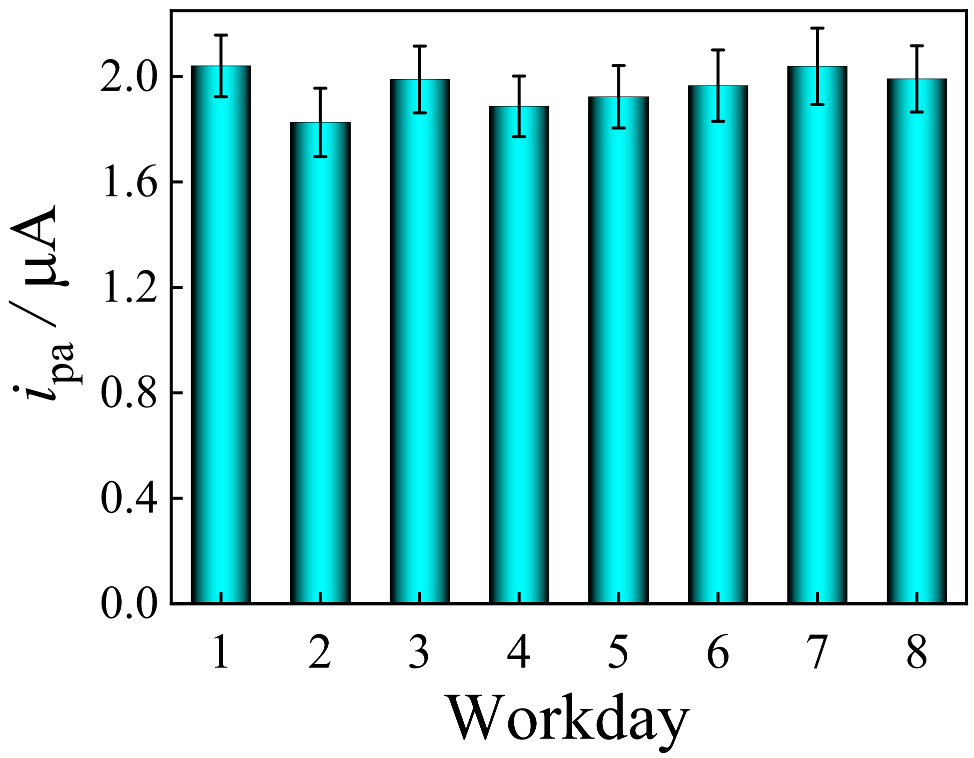

Figure S10. Voltammetric signal for 100 μmol L^{-1} SA measured over eight workdays. The bar height corresponds to the average value from three measurements and the error bars represent the standard deviation.

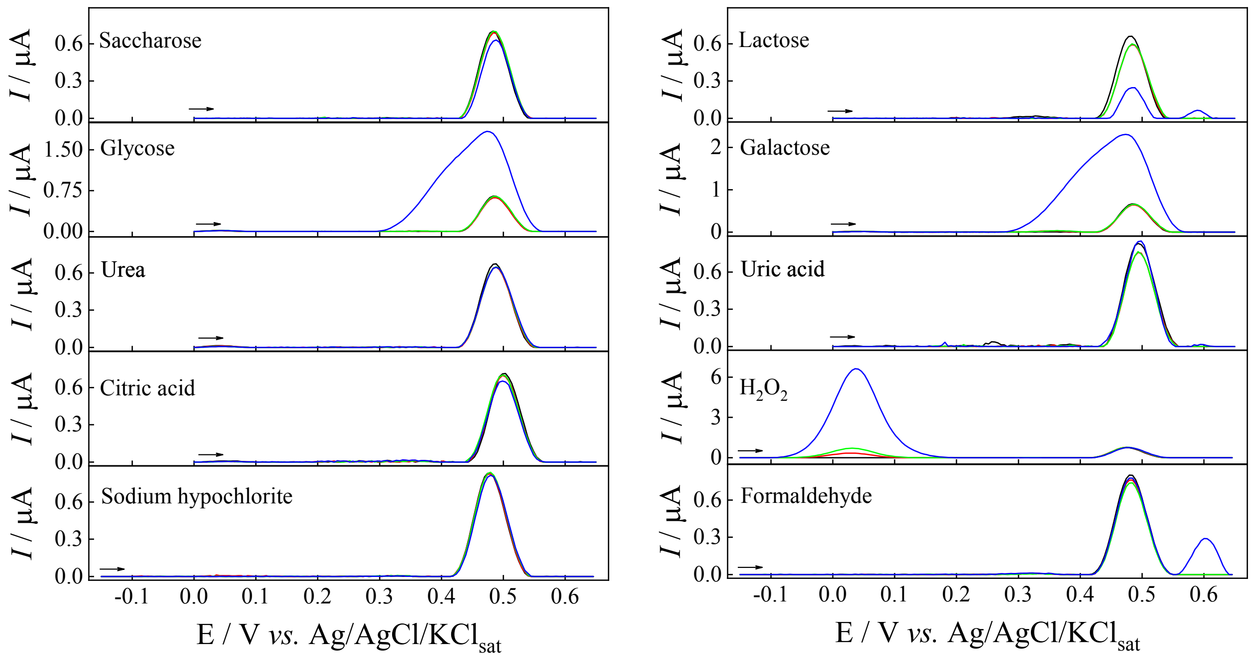

Figure S11. Baseline corrected differential pulse voltammograms recorded with a preconditioned Cu wire electrode (Ø = 1.3 mm) in 0.1 mol L^{-1} NaOH in the presence of 100 µmol L^{-1} SA and 100 µmol L^{-1} SA + varying concentrations of the interfering species. Voltammetric conditions: pulse amplitude = 50 mV; pulse width = 25 ms; and step potential = 5 mV. (—) 100 µmol L^{-1} SA. (—) 100 µmol L^{-1} SA + 50 µmol L^{-1} interfering species. (—) 100 µmol L^{-1} SA + 100 µmol L^{-1} interfering species. (—) 100 µmol L^{-1} SA + 1000 µmol L^{-1} interfering species.

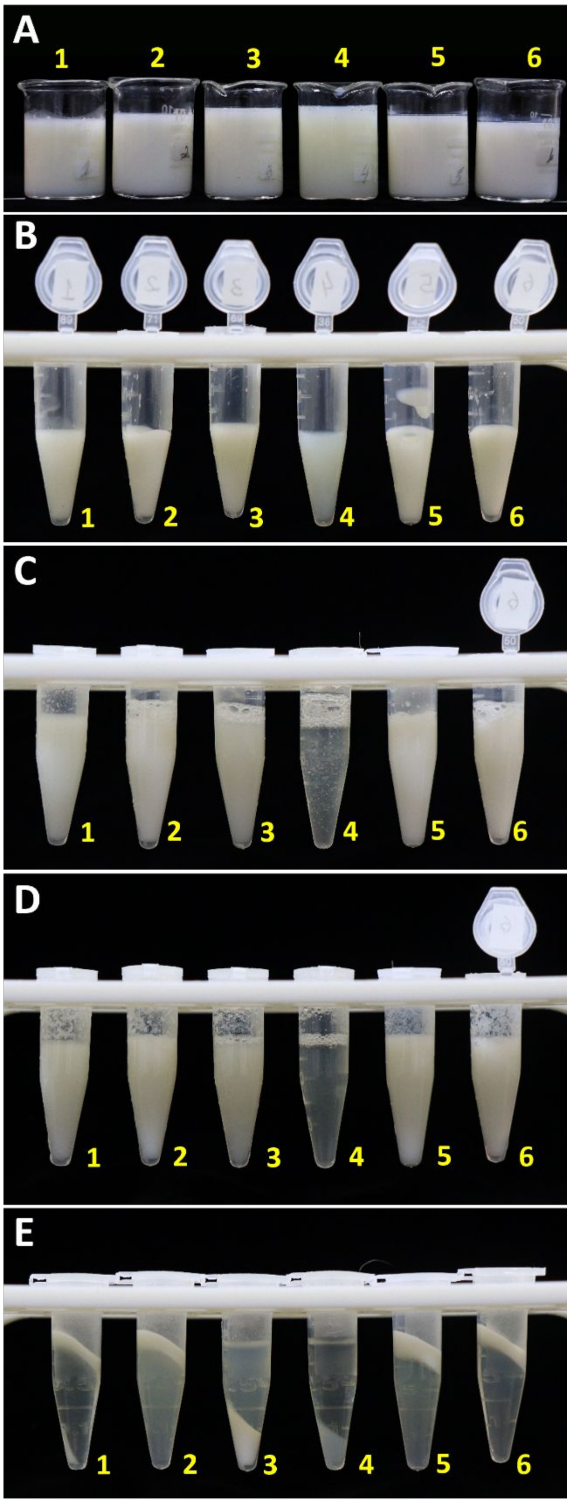

Figure S12. Representative images illustrating the steps of the milk sample pretreatment procedure. A) Untreated milk samples. B) 500 µL aliquots of milk transferred to polypropylene microcentrifuge tubes. C) Samples after the addition of 100 µL of 15 % (w/v) $ZnSO_4$ and 400 µL of 2.5 mol L^{-1} NaOH aqueous solutions. D) Samples after 10 min of sonication. E) Final extracts obtained after centrifugation at 12,000 rpm for 5 min. Milk samples: 1 – Raw, 2 – Whole, 3 – Semi-skimmed, 4 – Skimmed, 5 – Multivitamin-fortified, and 6 – Whole lactose-free.

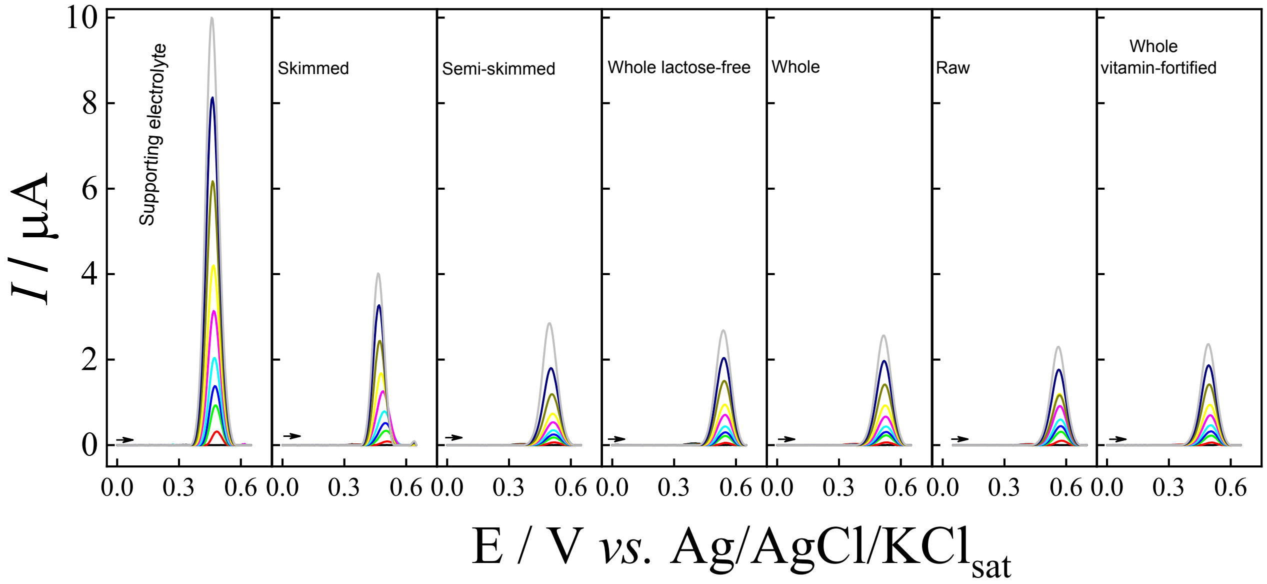

Figure S13. Baseline corrected differential pulse voltammograms recorded with a preconditioned Cu wire electrode (*Ø* = 1.3 mm) in the final milk extracts. Voltammetric conditions: ΔE = 50 mV; pulse width = 25 ms, and step potential = 5 mV. SA concentrations: (—) 0; (—) 10; (—) 20; (—) 50; (—) 70; (—) 100; (—) 150; (—) 200; (—) 300; (—) 400, and (—) 500 µmol L^{-1}. The baselines were corrected using the moving average mode with window size of 2.

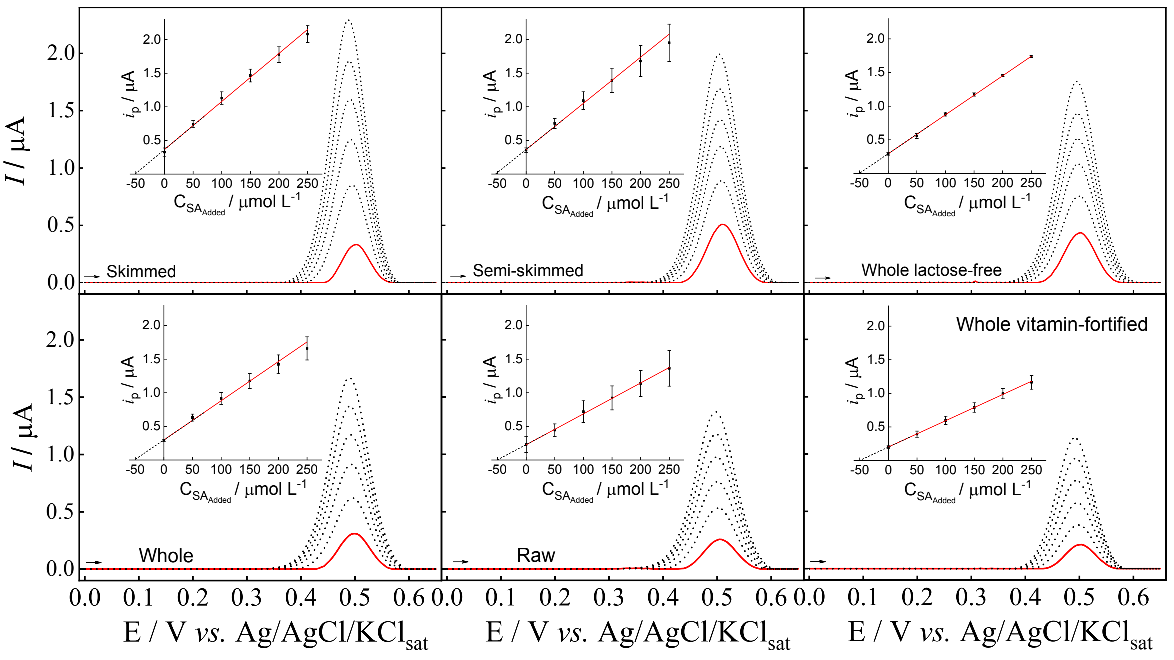

Figure S14. Baseline-corrected differential pulse voltammograms recorded with a preconditioned Cu wire electrode (Ø = 1.3 mm) in the final milk extracts. (—) Signal from the spiked sample. (---) Successive standard additions of 50 µmol L^{-1} SA. Voltammetric conditions: ΔE = 50 mV; pulse width = 25 ms, and step potential = 5 mV. Insets show the corresponding standard addition curves. Error bars represent the standard deviation of i_{pa} (n = 3). Baselines were corrected using the moving average mode with window size of 2.

Table S1. Evaluated ranges and selected experimental parameters for differential pulse voltammetry (DPV) and square wave voltammetry (SWV).

Technique	Parameter	Evaluated range	Selected value
SWV	Frequency, f (Hz)	10 – 100	50
	Pulse amplitude, ΔE (mV)	10 – 150	25
	Step potential, ΔE_s (mV)	2 – 6	4
DPV	Pulse amplitude, ΔE (mV)	25 – 150	50
	Pulse width, (ms)	5 – 100	25
	Step potential (mV)	2 – 6	5

Table S2. Analytical performance of some electrochemical methods for SA determination.

Electrode	Technique	Linear range (µmol L^{-1})	LOD (µmol L^{-1})	Sample	Ref.
PTFE/RGO film	DPV	0.0001–10	2.3×10^{-5}	Pharmaceutical formulations	1
Cu-MOFs-CB-nafion/SPE	DPV	100–900	12.5	Cucumber seedlings	2
MIPs/TiO_2	DPV	0.1–50	0.039	Pharmaceutical formulations	3
Ce/ZrO_2-CPE	SWV	5–1000	1.1	Milk; serum	4
CFE	DPV	2–3000	1.68	Pharmaceutical formulations	5
GCE	DPV	7–434	7	Pharmaceutical formulations	6
MWCNTs	Amperometry	2–3000	0.8	Pharmaceutical formulations	7
C-SPE	SWV	16–300	5.6	Urine	8
Carbon tape	DPV	0.5–100	0.05	Tomato leaves	9
Graphite-epoxy composite	DPV	5–200	5	Mouse blood	10
GCE-ZnO/CuO/Y_2O_3	DPV	1.5–15	0.18	Fruit juice	11
**Cu**	**DPV**	**10–500**	**3**	**Milk**	**This study**

PTFE: polytetrafluoroethylene, **rGO:** reduced graphene oxide, **MOF:** metal-organic framework, **CB:** carbon black, **SPE:** screen-printed electrode, **MIP**: molecularly imprinted polymer, **CPE:** carbon paste electrode, **GCE:** glassy carbon electrode, **MWCNTs:** multi-walled carbon nanotubes, **C-SPE:** carbon screen-printed electrode.

References

(1) Yu, Q.; Zhao, Y.; Huang, L.; Sun, J.; Jin, D.; Shu, Y.; Xu, Q.; Hu, X. Y. A Flexible RGO Electrode: A New Platform for the Direct Voltammetric Detection of Salicylic Acid. *Analytical Methods* **2020**, *12* (31), 3892–3900. https://doi.org/10.1039/d0ay00112k.

(2) Yang, L.; Chen, D.; Wang, X.; Luo, B.; Wang, C.; Gao, G.; Li, H.; Li, A.; Chen, L. Ratiometric Electrochemical Sensor for Accurate Detection of Salicylic Acid in Leaves of Living Plants. *RSC Adv* **2020**, *10* (64), 38841–38846. https://doi.org/10.1039/d0ra05813k.

(3) Xiong, X.; Li, C.; Yang, X.; Shu, Y.; Jin, D.; Zang, Y.; Shu, Y.; Xu, Q.; Hu, X.-Y. In Situ Grown TiO2 Nanorod Arrays Functionalized by Molecularly Imprinted Polymers for Salicylic Acid Recognition and Detection. *Journal of Electroanalytical Chemistry* **2020**, *873*, 114394. https://doi.org/10.1016/j.jelechem.2020.114394.

(4) Alizadeh, T.; Nayeri, S. Electrocatalytic Oxidation of Salicylic Acid at a Carbon Paste Electrode Impregnated with Cerium-Doped Zirconium Oxide Nanoparticles as a New Sensing Approach for Salicylic Acid Determination. *Journal of Solid State Electrochemistry* **2018**, *22* (7), 2039–2048. https://doi.org/10.1007/s10008-018-3907-1.

(5) Park, J.; Eun, C. Electrochemical Behavior and Determination of Salicylic Acid at Carbon-Fiber Electrodes. *Electrochim Acta* **2016**, *194*, 346–356. https://doi.org/10.1016/j.electacta.2016.02.103.

(6) Torriero, A. A. J.; Luco, J. M.; Sereno, L.; Raba, J. Voltammetric Determination of Salicylic Acid in Pharmaceuticals Formulations of Acetylsalicylic Acid. *Talanta* **2004**, *62* (2), 247–254. https://doi.org/10.1016/j.talanta.2003.07.005.

(7) Zhang, W. De; Xu, B.; Hong, Y. X.; Yu, Y. X.; Ye, J. S.; Zhang, J. Q. Electrochemical Oxidation of Salicylic Acid at Well-Aligned Multiwalled Carbon Nanotube Electrode and Its Detection. *Journal of Solid State Electrochemistry* **2010**, *14* (9), 1713–1718. https://doi.org/10.1007/s10008-010-1014-z.

(8) Rawlinson, S.; McLister, A.; Kanyong, P.; Davis, J. Rapid Determination of Salicylic Acid at Screen Printed Electrodes. *Microchemical Journal* **2018**, *137*, 71–77. https://doi.org/10.1016/j.microc.2017.09.019.

(9) Sun, L. J.; Feng, Q. M.; Yan, Y. F.; Pan, Z. Q.; Li, X. H.; Song, F. M.; Yang, H.; Xu, J. J.; Bao, N.; Gu, H. Y. Paper-Based Electroanalytical Devices for in Situ Determination of Salicylic Acid in Living Tomato Leaves. *Biosens Bioelectron* **2014**, *60*, 154–160. https://doi.org/10.1016/j.bios.2014.04.021.

(10) Zhu, Y.; Guan, X.; Ji, H. Electrochemical Solid Phase Micro-Extraction and Determination of Salicylic Acid from Blood Samples by Cyclic Voltammetry and Differential Pulse Voltammetry. *Journal of Solid State Electrochemistry* **2009**, *13* (9), 1417–1423. https://doi.org/10.1007/s10008-008-0707-z.

(11) Aljabri, M. D.; Mahmud Alam, M.; Fazle Rabbee, M.; Ahmed, J.; Al-Humaidi, J. Y.; Abdel-Fadeel, M. A.; Almahri, A.; Rahman, M. M. Electrochemical Detection of Salicylic Acid in Fruit Products with Ternary ZnO/CuO/Y2O3 Nanocomposites Embedded PEDOT:PSS by Differential Pulse Voltammetry. *Measurement* **2025**, *240*, 115583. https://doi.org/10.1016/j.measurement.2024.115583.
